# Exploring the Helical Structure of Ethylene Oxides: Beyond Steric and Related Effects

**DOI:** 10.1002/cphc.202500458

**Published:** 2025-08-27

**Authors:** Matheus P. Freitas

**Affiliations:** ^1^ Department of Chemistry Institute of Natural Sciences Federal University of Lavras Lavras MG 37200‐900 Brazil

**Keywords:** conformational analysis, ethylene oxides, helicity, hyperconjugation, solvation effects

## Abstract

Dimethoxyethane (DME) is the monomer of polyethylene oxide, a polymer widely used in materials science, with its conformation affecting properties such as host–guest interactions with ions. This quantum‐chemical study reveals that DME undergoes rotational isomerization among nine rotamers, favoring a *zigzag* (all‐*trans*) conformation, followed by a conformer with the OCCO fragment in a *gauche* arrangement. This preference strengthens with increasing chain length but transitions to a *gauche* configuration resembling a helical structure in polar solvents like DMSO. Unlike expectations based on steric effects or earlier assumptions of a 1,5 CH_3_/O nonbonding attraction, the helical structure arises from reduced dipolar repulsion/dipole stabilization and hyperconjugative interactions that surpass Lewis‐type interactions. The helical structure of a 21‐heavy‐atom oligomer further compresses to encapsulate a potassium cation, forming a highly stable complex.

## Graphical abstract

A helical structure in ethylene oxides begins to emerge early as the chain length increases in solution. This formation is facilitated by the attenuation of dipolar repulsion and stabilization of dipoles. However, it is primarily driven by hyperconjugative interactions, which often underpin *gauche* effects and dominate over Lewis‐type interactions.

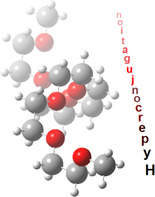

## 1 Introduction

Ethylene oxides are versatile molecules with significant implications in material science, polymer chemistry, and industrial applications. Their conformational behavior directly impacts the physical and chemical properties of derived polymers and materials, such as polyethylene oxide (PEO). The conformational behavior of 1,2‐dimethoxyethane (DME) and triglyme has been extensively studied both computationally and via NMR spectroscopy.^[^ ^1^, ^2^, ^3^, ^4^, ^5^, ^6^ ^]^ For DME, the linear *zigzag* (all‐*trans*, *ttt*) conformer was found to be the most stable, with an energy advantage of 0.2 kcal mol^−1^ over the second most stable conformer, the σ_3_ structure (a *tgt* conformer), among ten possibilities.^[^ ^1^ ^]^ In solution, the *gauche* conformer is preferred, while in the gas phase, the *tgg′* conformation appears to be stabilized by O···H interactions, particularly a 1,5 CH_3_/O nonbonding attraction.^[^ ^2^, ^3^ ^]^ In contrast, triglyme exhibits a weak *gauche* effect, with its most stable conformer adopting an arrangement where all 11 four‐heavy atom fragments are in a *gauche* orientation (5 *g^+^* and 6 *g^−^*). Two other conformers, containing 6 and 7 *gauche* fragments, are also more stable than the all‐*trans* conformer.^[^ ^1^ ^]^

Perfluoroalkanes, on the other hand, tend to favor helical conformations due to hyperconjugative interactions. This preference becomes more pronounced with increasing chain length, reaching an energetic stabilization of 3–5 kcal mol^−1^ for perfluorodecane.^[^ ^7^ ^]^ In a related context, the conformation of (PEO, with molecular weights ranging from 300 000 to 600 000 g mol^−1^, significantly impacts properties like solubility and rheology, especially in combination with surfactants.^[^ ^8^ ^]^ The conformational landscape of PEO also plays a crucial role in material sciences, influencing areas such as clay science^[^ ^9^, ^10^ ^]^ and ionic liquid studies.^[^ ^11^ ^]^ Notably, PEO adopts a helical conformation starting with six ethylene oxide units, facilitating host–guest interactions with alkali metal ions, particularly K^+^.^[^ ^12^ ^]^ This helical structure has also been supported by molecular dynamics simulations.^[^ ^13^, ^14^, ^15^ ^]^ Given the broader implications, understanding the conformational behavior of model ethylene oxides provides insights into modulating and predicting PEO properties.

**Figure 1:**
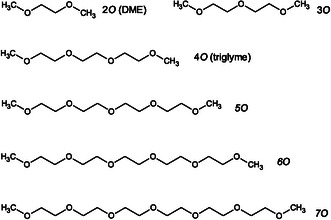
Structures of ethylene oxides analyzed in this study using quantum‐chemical calculations.

To explore how the conformational preferences of ethylene oxides evolve toward a helical structure—or deviate from it—based on chain length, a quantum‐chemical theoretical study is performed. This study will focus on monomeric DME (6 heavy atoms) and oligomers containing 9, 12, 15, 18, and 21 heavy atoms (**Figure** **1**). Additionally, natural bond orbital (NBO) analysis will be applied to selected conformations to assess the contributions of hyperconjugative effects (often linked to the *gauche* effect in 1,2‐disubstituted ethanes like 1,2‐difluoroethane^[^ ^16^, ^17^, ^18^, ^19^, ^20^ ^]^) and Lewis‐type interactions, including steric effects, in shaping the conformational landscape.

## 2 Computational Methods

The 27 possible staggered conformations of DME were fully optimized with frequency calculations at both the G3MP2B3 and B3LYP‐GD3BJ/6‐311++G(d,p) levels of theory.^[^ ^21^, ^22^, ^23^, ^24^, ^25^ ^]^ The G3MP2B3 composite method involves initial geometry optimization at the B3LYP/6‐31 G(d) level, followed by a QCISD(T,FC)/6‐31 G(d)//B3LYP/6‐31 G(d) single‐point calculation, and further energy refinement via a single‐point MP2(FC)/G3MP2large//B3LYP/6‐31G(d) calculation. In contrast, the B3LYP‐GD3BJ/6‐311++G(d,p) level is less computationally demanding while still yielding accurate conformational energies for small systems.^[^ ^19^ ^]^ Calculations were carried out in both the gas phase and implicit dimethyl sulfoxide (DMSO) solution using the solvation model density (SMD) approach.^[^ ^26^ ^]^ From these calculations, nine nonequivalent conformers were identified: *ttt*, *tgt*, *ttg*, *tgg*, *tgg′*, *gtg*, *gtg′*, *ggg*, and *ggg′*. Here, *t*, *g*, and *g′* denote the *trans*, *gauche* (+60°), and *gauche’* (−60°) dihedral angles of the COCC, OCCO, and CCOC moieties. Given the similar results from both levels of theory and the computational efficiency of the density functional theory (DFT) approach, this method was applied to analyze ethylene oxides containing 3–7 oxygen atoms (3*O*, 4*O*, 5*O*, 6*O*, and 7*O*). The conformations selected for these calculations were *tt*_*n*_ *t* and *tg*_*n*_ *t*, corresponding to the two most stable conformers of DME. The variable *n* represents the number of OCCO orientations, ranging from 2 to 6—for example, the *tg*_*n*_ *t* conformer with *n* = 3 (3*O*) corresponds to a *tggt* conformation, *tgggt* for 4*O*, and so forth. To further explore electron delocalization, second‐order perturbation analysis of donor−acceptor interactions within the NBOs^[^ ^27^ ^]^ was conducted. Using the NBODEL NOSTAR keywords, deletion energy was computed by localizing electrons in a perfect Lewis structure, providing insights into electron delocalization contributions. Additionally, atoms‐in‐molecules (AIM) analyses were performed to identify intramolecular interactions in DME, particularly in the *tgg′* conformer, by examining the electron density at bond critical points along bond paths. Calculations were carried out using the Gaussian 16 suite of programs^[^ ^28^ ^]^ and AIMAll software.^[^ ^29^ ^]^

## 3 Results and Discussion

DME undergoes rotational isomerization around three dihedral angles: COCC, OCCO, and CCOC. These angles can adopt either *trans* (*t*) or *gauche* (*g* and *g*′, ±) conformations, resulting in 27 theoretical rotameric possibilities. Geometry optimizations of these rotamers in the gas phase were performed at the G3MP2B3 and B3LYP‐GD3BJ/6‐311++G(d,p) levels of theory,^[^ ^21^, ^22^, ^23^, ^24^, ^25^ ^]^ yielding nine nonequivalent structures. Given the comparable energy values obtained at both levels of theory, further analysis will focus on the results from the DFT level. Their relative standard Gibbs free energies are presented in **Table** **1**.

**Table 1:** Relative standard Gibbs free energies (in kcal mol^−1^) for the rotamers of ethylene oxides in the gas phase/implicit DMSO (SMD), obtained at the B3LYP‐GD3BJ/6‐311++G(d,p) level. The G3MP2B3 relative energies are given in parentheses.

Rotamer	2*O* (DME)	3*O*	4*O*	5*O*	6*O*	7*O*
*tt*_*n*_ *t* (*zigzag*)	0.0 (0.0)/0.6 (0.5)	0.0/0.9	0.0/1.3	0.0/1.5	0.0/1.8	0.0/3.1
*tg*_*n*_ *t* (*gauche* OCCO)	0.4 (0.3)/0.0 (0.0)	0.5/0.0	0.6/0.0	1.0/0.0	0.8/0.0	0.8/0.0
*ttg*	1.1 (1.3)/1.3 (1.3)	–	–	–	–	–
*tgg*	1.5 (1.6)/0.7 (0.8)	–	–	–	–	–
*tgg′*	0.3 (0.7)/0.9 (1.4)	–	–	–	–	–
*gtg*	2.4 (2.7)/1.9 (2.5)	–	–	–	–	–
*gtg′*	2.2 (2.5)/2.2 (2.5)	–	–	–	–	–
*ggg*	2.3 (2.3)/1.8 (1.5)	–	–	–	–	–
*ggg′*	1.7 (2.3)/1.9 (2.3)	–	–	–	–	–

Consistent with previous studies, the *zigzag* conformation of DME, denoted as *ttt*, was identified as the most stable structure, with a small energy difference of 0.3–0.4 kcal mol^−1^ relative to the next most stable rotamers, *tgt* and *tgg′*, characterized by an OCCO dihedral angle of ≈75.6° and 74.2°, respectively. Unlike the fluorines in 1,2‐difluoroethane, the methoxy groups in DME do not induce a *gauche* effect in the ethane fragment in the gas phase—a behavior expected to persist in nonpolar solution. A NBO analysis provides insights into the origin of this behavior in DME.

In the NBO framework, the full electronic energy of the systems can be decomposed into Lewis‐type (*E*_L_) and non‐Lewis‐type (*E*_NL_) interactions. Lewis‐type interactions encompass Pauli repulsion and dipolar repulsion, arising from a perfectly localized electron distribution within a Lewis structure; in contrast, non‐Lewis‐type interactions represent stabilization effects stemming from electron delocalization.^[^ ^27^ ^]^ The primary contributions to electron delocalization involve antiperiplanar interactions between electron donor and acceptor orbitals. As shown in **Table** **2**, the *ttt* conformation is the least disfavored by *E*_L_ due to weaker steric repulsion in the *trans* arrangement of the OCCO and COCC moieties, but it is the least favored by *E*_NL_ due to reduced electron delocalization. In contrast, the *ggg* conformation is the most disfavored by *E*_L_, while *tgg* is the most favored by *E*_NL_. For the *ttt* conformation, the small steric repulsion compensates for the low stabilization from electron delocalization, making it the most stable overall. Notably, the high stability of the *tgg′* and *tgt* conformations arises from a balance between intermediate *E*_L_ and *E*_NL_ contributions, with antiperiplanar hyperconjugative interactions between donor (*σ*_CH_) and acceptor (σ*_CO_) orbitals providing stabilization of 4.1 and 4.2 kcal mol^−1^ for *tgg′* and two of 3.6 kcal mol^−1^ for *tgt*.

**Table 2:** NBO analysis showing the decomposition of the relative full electronic energy (*E*_full_) into Lewis‐type (*E*_L_) and non‐Lewis‐type (*E*_NL_) components, expressed in kcal mol^−1^. Results are presented as *E*_full_/*E*_L_/*E*_NL_ for both the gas phase (first entries) and implicit DMSO (second entries).

Rotamer	2*O* (DME)	3*O*	4*O*	5*O*	6*O*	7*O*
*tt*_*n*_ *t*	0.0/213.3/−213.3	0.0/333.3/−333.3	0.0/453.2/−453.2	0.0/573.0/−573.0	0.0/693.1/−693.1	0.0/813.1/−813.1
	0.5/207.8/−207.3	1.1/325.9/−324.8	1.7/444.0/−442.3	2.2/562.1/−559.8	2.8/680.2/−677.3	3.4/798.3/−794.8
*tg*_*n*_ *t*	0.4/219.4/−219.0	0.5/344.9/−344.4	0.6/470.6/−470.0	0.7/596.3/−595.6	0.8/722.0/−721.2	1.0/847.9/−846.9
	0.0/212.7/−212.7	0.0/335.7/−335.7	0.0/458.8/−458.8	0.0/582.0/−582.0	0.0/705.1/−705.1	0.0/828.1/−828.1
*ttg*	1.3/215.3/−214.0	–	–	–	–	–
	1.4/209.1/−207.7	–	–	–	–	–
*tgg*	1.6/224.6/−223.0	–	–	–	–	–
	0.7/215.2/−214.5	–	–	–	–	–
*tgg′*	0.1/217.7/−217.6	–	–	–	–	–
	0.8/213.8/−213.0	–	–	–	–	–
*gtg*	2.7/217.6/−214.9	–	–	–	–	–
	2.3/209.8/−207.5	–	–	–	–	–
*gtg′*	2.5/216.7/−214.1	–	–	–	–	–
	2.3/210.4/−208.1	–	–	–	–	–
*ggg*	1.7/222.7/−221.1	–	–	–	–	–
	1.2/216.6/−215.3	–	–	–	–	–
*ggg′*	1.4/220.8/−219.4	–	–	–	–	–
	1.3/215.0/−213.7	–	–	–	–	–

**Figure 2:**
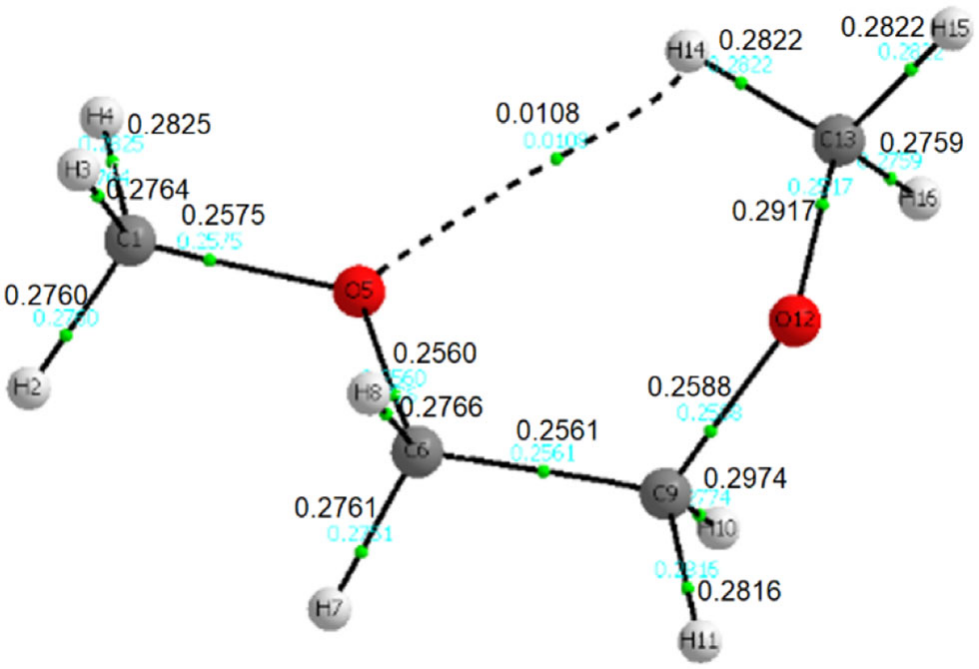
AIM plot for the *tgg′* conformer of DME, highlighting a bond path that stems from a 1,5 CH_3_/O nonbonding interaction.

Contrary to expectations based on steric considerations, the *tgg′* conformer is similarly populated to the *tgt* conformer, despite its *gauche* CCOC dihedral angle, which induces a butane‐*gauche*‐like interaction involving a methyl group. To elucidate the relatively small *E*_L_ term of *tgg′*, an AIM analysis was conducted. This analysis revealed a bond path with an electron density at the bond critical point of 0.0108 (**Figure** **2** **)**, supporting the presence of a 1,5 CH_3_/O nonbonding attraction that stabilizes the *tgg′* conformer, as previously reported.^[^ ^2^, ^3^ ^]^

**Figure 3:**
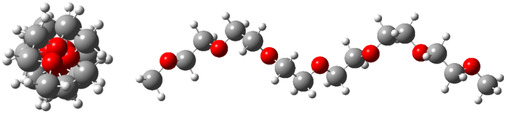
Front and side views of the helical structure of *tg*_*n*_ *t* 7*O*, optimized at the B3LYP‐GD3BJ/6‐311++G(d,p) level, illustrating the hydrophilic interior and lipophilic exterior characteristic of the helix.

The preference for the *zigzag* conformation strengthens as the chain extends from two to five oxygens, after which the energy difference relative to the *tg*_*n*_ *t* (*n* = number of OCCO *gauche* interactions, from 1 to 6) conformation stabilizes. This trend suggests that additional *gauche* units along the OCCO backbone lead to increased steric and dipolar repulsion, as reflected by the *E*_L_ term shown in Table 2. Notably, the *tg*_*n*_ *t* conformation adopts a helical structure, which becomes progressively more pronounced as the ethylene oxide chain lengthens, exemplified by the 7*O* oligomer containing six ethylene oxide units (**Figure** **3**). Therefore, the factors governing the conformational stability of DME are consistent with those of other ethylene oxides, underscoring the significance of investigating small model molecules to gain insights into the general characteristics of larger systems.

However, the conformational preferences shift when DME is placed in a continuous dielectric medium, such as the polar solvent DMSO, where real‐world phenomena like ion transport typically occur, rather than in the isolated state. Using a SMD to simulate DMSO, the *tgt* conformer emerges as the most stable, with the *ttt* conformer being 0.6 kcal mol^−1^ less stable, and the *tgg´* conformer 0.9 kcal mol^−1^ less stable than *tgt*, despite its higher dipole moment (1.72 D vs 1.45 D). This preference extends to larger ethylene oxides, suggesting that helicity emerges early in these systems, rendering any 1,5 CH_3_/O nonbonding interaction inactive. For 7*O*, the preference for the *tg*_*n*_ *t* conformation over the *tt*_*n*_ *t* conformation reaches 3.1 kcal mol^−1^, suggesting that in DMSO, *tg*_*n*_ *t* is almost the exclusive form. Given that the *zigzag* conformation of ethylene oxides with an even number of oxygens is nonpolar, a polar medium is expected to primarily stabilize the more polar *gauche* conformers by mitigating dipolar repulsion or stabilizing dipoles. This raises the question: after stabilizing dipolar interactions while maintaining steric effects, what interactions drive the helicity in ethylene oxides?

**Figure 4:**
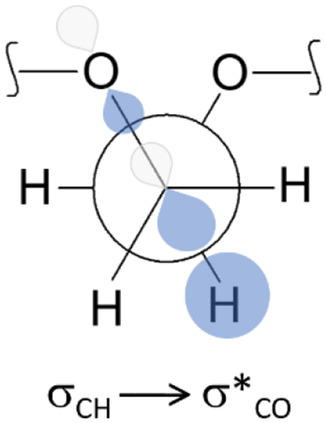
Hyperconjugative interaction responsible for the *gauche* effect in ethylene oxides.

Table 2 shows that the *ttt* conformation of DME in DMSO exhibits lower repulsive interactions (*E*_L_) compared to other conformations. However, this term becomes less significant in conformers with a *gauche* OCCO dihedral angle, particularly *tgt*, as dipolar repulsion, reflected in *E*_L_, diminishes in a polar solvent, thereby enhancing the role of electron delocalization (*E*_NL_). Generally, conformers with a *trans* OCCO arrangement are less stabilized by *E*_NL_, whereas those with a *gauche* OCCO dihedral angle promote hyperconjugative interactions, as depicted in **Figure** **4**. The greater the number of *gauche* OCCO arrangements in a structure, the larger the *E*_NL_ energy difference between *tt*_*n*_ *t* and *tg*_*n*_ *t* (helical) conformations, with this difference increasing with chain length. Specifically, *tg*_*n*_ *t* is more stabilized by E_NL_ than *tt*_*n*_ *t* by ≈5.4, 10.9, 16.5, 22.2, 27.8, and 33.3 kcal mol^−1^ for DME, 3*O*, 4*O*, 5*O*, 6*O*, and 7*O*, respectively. Thus, the *gauche* effect originated from *σ*_CH_ → σ*_CO_ interactions intensifies with increasing chain size under conditions of dipole stabilization or minimized dipolar repulsion. A geometric indication of this effect in a polar solution is the reduction of the OCCO dihedral angle in *tg*_*n*_ *t*, which decreases from 74.3°–74.9° for 7*O* in the isolated state to 71.6°–72.2° in implicit DMSO.

**Figure 5:**
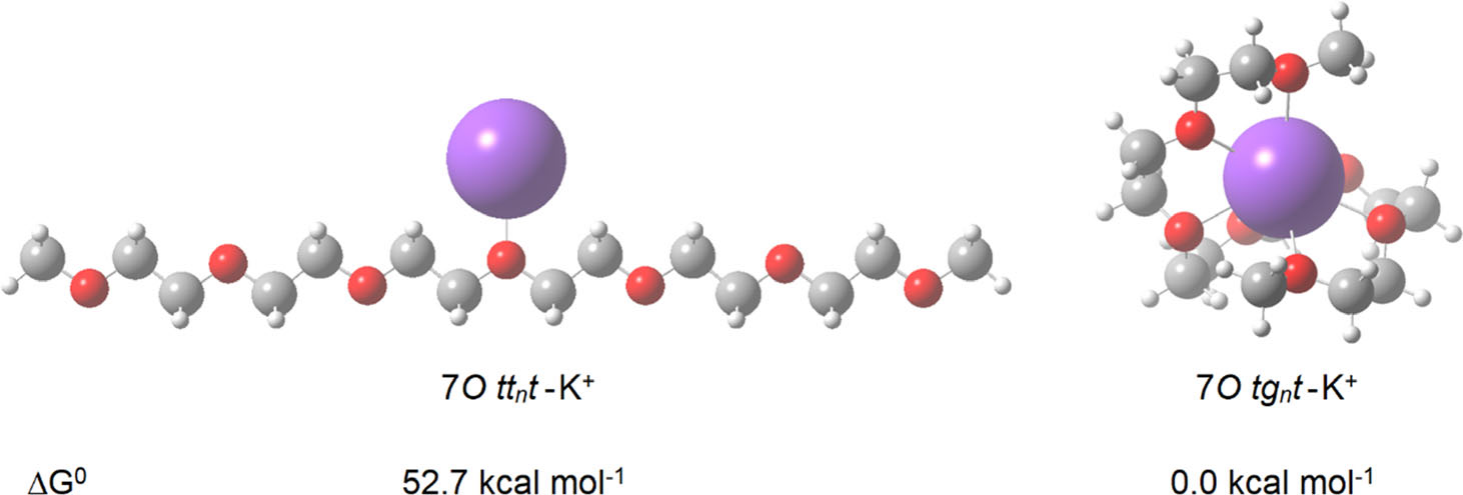
Mono‐ and hepta‐coordinated complexes of 7*O* with a potassium cation, optimized with counterpoise correction, starting from the *zigzag* (left) and *tg*_*n*_ *t* (right) conformations. The potassium ion in the input geometry was positioned near the central oxygen at a distance of 2.7 Å. The relative standard Gibbs free energies of the gas‐phase conformations, calculated at the B3LYP‐GD3BJ/6‐311++G(d,p) level, are provided.

Considering the helical conformation of PEO as the preferred structure in solution, characterized by a hydrophilic interior and a lipophilic exterior, it can effectively bind cations for transport purposes. A simulation performed at the B3LYP‐GD3BJ/6‐311++G(d,p) level, starting from the *tg*_*n*_ *t* conformation of 7*O* with a potassium cation positioned near the central oxygen, revealed that the helix compresses to maximize interactions between K^+^ and the oxygens. This optimization resulted in a highly stable hepta‐coordinated complex (**Figure** **5**). The stability of the complex was evaluated by comparing its energy to that of the isolated species (K^+^ and 7*O*), yielding a complexation enthalpy (Δ*H* ^0^) of −82.9 kcal mol^−1^. In contrast, the corresponding value for the *zigzag* conformation was significantly lower, at −21.3 kcal mol^−1^. These findings suggest that, much like crown ethers, ethylene oxides merit exploration as ion carriers due to their cylindrical shape and amphiphilic properties.

Although the complexation is exothermic, the selectivity of the helical structure of 7*O* toward different alkali metals (Li^+^ and Na^+^) does not align with the experimental trend observed by mass spectrometry.^[^ ^12^ ^]^ Computational results indicate that the complexation affinity follows the order Li^+^ (−123.3 kcal mol^−1^) > Na^+^ (−103.3 kcal mol^−1^) > K^+^ (−82.9 kcal mol^−1^), which is the reverse of what is observed experimentally. Our simulations suggest that the helical structure of 7*O* can adapt its cavity to accommodate the alkali metal ions, allowing smaller ions—whose size is closer to that of the oxygen atoms—to interact more effectively with all the oxygen atoms overall.

## 4 Conclusion

In the gas phase, DME and higher ethylene oxides adopt a *zigzag* conformation due to reduced steric and dipolar effects. However, in polar solution, where dipoles are stabilized, a *gauche* orientation of the OCCO dihedral angle predominates, leading to a helical structure that becomes more apparent as the ethylene oxide chain length increases. Unlike in the gas phase, non‐Lewis contributions from electron delocalization surpass Lewis‐type interactions in implicit DMSO. This is driven by *σ*_CH_ → σ*_CO_ hyperconjugation, involving antiperiplanar donor and acceptor orbitals, analogous to the *gauche* effect observed in 1,2‐difluoroethane. The helical structure of ethylene oxides features a polar cavity with a hydrophobic exterior, enabling interactions with cations and facilitating their transport in organic media, similar to crown ethers. The simulation of a system containing six ethylene oxide units (7*O*) revealed that complexation with a potassium cation is highly favorable, underscoring its suitability as an ion carrier. Thus, the *gauche* effect in ethylene oxides arises in environments reflective of real‐world phenomena, dictating the helical conformation critical to their functional potential.

## Supporting Information

Optimized structures' standard Cartesian coordinates at the G3MP2B3 and B3LYP‐GD3BJ/6‐311++G(d,p) levels.

## Conflict of Interest

The authors declare no conflict of interest.

## Author Contributions

**Matheus P. Freitas**: conceptualization (lead); formal analysis (lead); funding acquisition (lead); investigation (lead); methodology (lead); writing—original draft (lead); writing—review and editing (lead).

## Supporting information

Supplementary Material

## Data Availability

The data that support the findings of this study are available in the supplementary material of this article.
