# Supplementary material for: Exploring the Helical Structure of Ethylene Oxides: Beyond Steric and Related Effects
Source: Chemphyschem. 2025 Aug 27;26(20):e202500458. doi: 10.1002/cphc.202500458 (PMC12530874; doi:10.1002/cphc.202500458)
Supplement: Supplementary file 1 — Supplementary Material [file CPHC-26-e202500458-s001.pdf]

SUPPLEMENTARY INFORMATION

Exploring the Helical Structure of Ethylene Oxides: Beyond Steric and Related Effects

Matheus P. Freitas*[a]

[a] Department of Chemistry, Institute of Natural Sciences, Federal University of Lavras, 37200-900, Lavras, MG, Brazil
E-mail: matheus@ufla.br

Pages S2-S61: Standard coordinates and standard Gibbs free energies of the optimized ethylene oxides.

DME *ttt* (gas phase, G3MP3B3)

0 1

C 2.98082100 -0.11366400 0.00011900

H 3.12110500 -0.74430300 0.89314400

H 3.12132900 -0.74461400 -0.89265100

H 3.73813600 0.67483300 0.00007200

O 1.71988200 0.51910400 -0.00014300

C 0.64660100 -0.40062500 -0.00009500

H 0.68010100 -1.05191700 0.88891500

H 0.67995900 -1.05184900 -0.88916100

C -0.64660100 0.40062500 0.00003000

H -0.68006000 1.05196800 -0.88894400

H -0.68000100 1.05179800 0.88913200

O -1.71988200 -0.51910400 -0.00002500

C -2.98082100 0.11366400 0.00005100

H -3.12118200 0.74463200 -0.89272900

H -3.73813600 -0.67483300 -0.00013300

H -3.12125200 0.74428500 0.89306600

G^0 = -308.415429 hartrees

DME *tgt* (gas phase, G3MP3B3)

0 1

C -2.74286100 -0.44372000 -0.06402000

H -3.32684800 0.46237100 0.17005700

H -2.80458600 -0.62143000 -1.15002200

H -3.18614600 -1.29478100 0.45968400

O -1.40709300 -0.33536900 0.37341100

C -0.72137400 0.74015300 -0.23257900

H -1.18913200 1.70279100 0.04125400

H -0.74886400 0.65539800 -1.33167200

C 0.72137400 0.74015400 0.23258000

H 0.74886400 0.65539500 1.33167300

H 1.18913100 1.70279200 -0.04125000

O 1.40709400 -0.33536700 -0.37341400

C 2.74286000 -0.44372100 0.06402100

H 2.80458100 -0.62145200 1.15002000

H 3.18615100 -1.29477000 -0.45969800

H 3.32684600 0.46237700 -0.17003500

G^0 = -308.414881 hartrees

DME *ttg* (gas phase, G3MP3B3)

0 1

C 2.82886100 0.38216200 0.03573600

H 2.74654000 1.44499100 -0.24486500

H 3.01995000 0.32563100 1.11960400

H 3.67431500 -0.06084000 -0.49742500

O 1.67677900 -0.34554700 -0.33038400

C 0.50235100 0.12970500 0.29712900

H 0.32058400 1.18483400 0.02996700

H 0.58810400 0.07856200 1.39515600

C -0.65416400 -0.75766900 -0.16504200

H -0.45336900 -1.78997300 0.13899600

H -0.71930600 -0.73626500 -1.26398900

O -1.88586600 -0.38946300 0.43106100

C -2.55272400 0.67334700 -0.22120600

H -2.75137100 0.43622800 -1.27860400

H -3.50654700 0.81216400 0.29496600

H -1.99214900 1.61947300 -0.17892900

G^0 = -308.413433 hartrees

DME *tgg* (gas phase, G3MP3B3)

0 1

C 2.59652700 -0.52222000 -0.00328200

H 3.18659200 0.22882300 0.54818200

H 2.43992500 -1.38962100 0.65911600

H 3.16463100 -0.84465700 -0.87987200

O 1.37610900 0.00769300 -0.46971700

C 0.54290500 0.46303600 0.57428400

H 1.03031400 1.27990400 1.13784100

H 0.34497700 -0.34901000 1.29530800

C -0.75620200 0.99403000 -0.02107200

H -0.50871300 1.72158600 -0.80092600

H -1.32081900 1.51567600 0.77154300

O -1.56362600 0.01604600 -0.64763500

C -2.27962400 -0.80796600 0.24730900

H -2.90782900 -0.21393700 0.93211900

H -2.92615600 -1.44688500 -0.36021500

H -1.62441900 -1.45306600 0.85228300

G^0 = -308.412928 hartrees

DME *tgg’* (gas phase, G3MP3B3)

0 1

C 2.48082800 0.51181300 -0.10942200

H 3.14060200 -0.30020400 0.23764700

H 2.55504300 0.57094000 -1.20722200

H 2.82396900 1.45601800 0.32163300

O 1.14877600 0.31720500 0.31576200

C 0.58942800 -0.88980400 -0.17213300

H 1.17037000 -1.75443100 0.19784400

H 0.61770300 -0.91498700 -1.27420700

C -0.85441200 -1.02430800 0.29575800

H -0.90077200 -0.84231700 1.38196600

H -1.17605100 -2.05615100 0.10977900

O -1.77462700 -0.20362500 -0.39633500

C -1.82466800 1.14195300 0.05024900

H -0.88976300 1.67921800 -0.14526600

H -2.64426000 1.61951000 -0.49443800

H -2.03709400 1.19584400 1.13014100

G^0 = -308.414271 hartrees

DME *gtg* (gas phase, G3MP3B3)

0 1

C 2.39158900 0.75461800 -0.18610300

H 1.87770900 1.23920400 -1.03005800

H 2.36413600 1.44082000 0.67576900

H 3.43421800 0.58375800 -0.46751500

O 1.84378600 -0.50758900 0.13943600

C 0.49722300 -0.46241500 0.58516900

H 0.33433100 0.40826200 1.24026900

H 0.33408400 -1.36456300 1.18367400

C -0.49722300 -0.46241100 -0.58517100

H -0.33408400 -1.36455500 -1.18368200

H -0.33433200 0.40827000 -1.24026600

O -1.84378600 -0.50759000 -0.13943700

C -2.39159000 0.75461800 0.18610500

H -2.36416000 1.44081200 -0.67577300

H -3.43421000 0.58375400 0.46754200

H -1.87769200 1.23921300 1.03004400

G^0 = -308.411172 hartrees

DME *gtg’* (gas phase, G3MP3B3)

0 1

C -2.65439600 -0.35152500 0.08332400

H -2.35048800 -1.37434300 -0.18513500

H -2.83265800 -0.31749400 1.16994400

H -3.58878700 -0.12067900 -0.43543700

O -1.71307400 0.62555800 -0.31736200

C -0.45503600 0.52281500 0.33072200

H -0.57407000 0.28113800 1.39860100

H 0.00029900 1.51661800 0.26238200

C 0.45503300 -0.52282100 -0.33069800

H 0.57405800 -0.28115200 -1.39857900

H -0.00029000 -1.51662900 -0.26234800

O 1.71307400 -0.62554000 0.31738200

C 2.65439800 0.35151900 -0.08336000

H 2.35047900 1.37435400 0.18501900

H 3.58878100 0.12071500 0.43543400

H 2.83267800 0.31740900 -1.16997400

G^0 = -308.411412 hartrees

DME *ggg* (gas phase, G3MP3B3)

0 1

C -2.07697400 0.59898100 -0.41732800

H -1.48519300 0.88446100 -1.29760300

H -2.72735200 -0.25069700 -0.68387400

H -2.70661000 1.44519000 -0.12934200

O -1.26212300 0.30165800 0.69986200

C -0.50850600 -0.88663500 0.57599900

H -1.16661800 -1.76557100 0.44615200

H 0.00085400 -1.00069800 1.53850500

C 0.50850700 -0.88664000 -0.57599100

H -0.00085200 -1.00071300 -1.53849700

H 1.16661900 -1.76557600 -0.44613700

O 1.26212500 0.30165100 -0.69986600

C 2.07697100 0.59898600 0.41732400

H 2.72734700 -0.25068900 0.68388300

H 2.70661000 1.44519100 0.12933000

H 1.48518700 0.88447800 1.29759300

G^0 = -308.411770 hartrees

DME *ggg'* (gas phase, G3MP3B3)

0 1

C -2.13302100 -0.62554300 0.40985800

H -1.62761900 -0.79623500 1.37248400

H -2.90881200 0.14306500 0.55850300

H -2.61296900 -1.55765400 0.09992100

O -1.23869600 -0.26148700 -0.62229000

C -0.58011700 0.98000400 -0.40793000

H -1.29548700 1.72744100 -0.02203700

H -0.23774300 1.31321600 -1.39355900

C 0.63100600 0.91538500 0.52606600

H 0.36343100 0.42533000 1.47784800

H 0.93262600 1.94425600 0.75967900

O 1.77050500 0.30334600 -0.04612300

C 1.73256100 -1.11403400 -0.07323600

H 0.94963700 -1.49675500 -0.73798900

H 2.71139800 -1.44500800 -0.43191100

H 1.56848600 -1.52739600 0.93581300

G^0 = -308.411839 hartrees

DME *ttt* (DMSO – SMD, G3MP3B3)

0 1

C 2.98186900 -0.11695100 0.00006000

H 3.11856300 -0.74808000 0.89271100

H 3.11864500 -0.74816200 -0.89252100

H 3.74752700 0.66492000 0.00005700

O 1.72155200 0.52675200 -0.00002600

C 0.64869300 -0.39689600 -0.00003500

H 0.68863900 -1.04786000 0.88894500

H 0.68858700 -1.04778600 -0.88907300

C -0.64869300 0.39689600 0.00003200

H -0.68861300 1.04789500 -0.88892500

H -0.68861400 1.04775100 0.88909300

O -1.72155200 -0.52675200 -0.00004500

C -2.98186900 0.11695100 -0.00000900

H -3.11857800 0.74821900 -0.89255900

H -3.74752700 -0.66492000 -0.00011700

H -3.11862900 0.74802200 0.89267300

G^0 = -308.420681 hartrees

DME *tgt* (DMSO – SMD, G3MP3B3)

0 1

C 2.76619200 -0.41337000 0.05681500

H 3.31523600 0.49775100 -0.23218400

H 2.86177000 -0.53991700 1.14735300

H 3.22618000 -1.27371000 -0.43900700

O 1.41282700 -0.36467900 -0.35113700

C 0.72061300 0.72699500 0.23086100

H 1.18584300 1.68244900 -0.06583100

H 0.75729600 0.66911100 1.33143600

C -0.72061300 0.72699500 -0.23086100

H -0.75729600 0.66911100 -1.33143600

H -1.18584300 1.68244900 0.06583100

O -1.41282700 -0.36468000 0.35113700

C -2.76619300 -0.41337000 -0.05681500

H -2.86177000 -0.53991700 -1.14735300

H -3.22618000 -1.27371100 0.43900700

H -3.31523600 0.49775100 0.23218400

G^0 = -308.421458 hartrees

DME *ttg* (DMSO – SMD, G3MP3B3)

0 1

C 2.82118600 0.39539100 0.03652100

H 2.72794000 1.44245100 -0.29328500

H 2.99853100 0.39082500 1.12384600

H 3.68305700 -0.05698100 -0.46356900

O 1.68166900 -0.36817300 -0.31267500

C 0.49562900 0.13053900 0.28301000

H 0.32361100 1.17604700 -0.01964000

H 0.56894300 0.11193300 1.38305800

C -0.65629900 -0.76651600 -0.16717600

H -0.45101300 -1.79555200 0.14897000

H -0.72751800 -0.75797300 -1.26597700

O -1.89394800 -0.40141400 0.42514600

C -2.53511300 0.69131700 -0.21544300

H -2.71041600 0.48576200 -1.28304300

H -3.50145300 0.82770300 0.27980200

H -1.96586200 1.62809500 -0.13140300

G^0 = -308.419330 hartrees

DME *tgg* (DMSO – SMD, G3MP3B3)

0 1

C 2.60068100 -0.51434500 0.00029200

H 3.18240100 0.25474700 0.53438700

H 2.45442600 -1.36798700 0.68193700

H 3.17649500 -0.85154500 -0.86726800

O 1.37117900 -0.00672100 -0.47969800

C 0.54125100 0.46232000 0.56952900

H 1.03521900 1.28232300 1.11989000

H 0.34886600 -0.34227900 1.29801300

C -0.75818200 0.99779800 -0.01250900

H -0.52079800 1.74188800 -0.78129300

H -1.31741300 1.50464400 0.79131000

O -1.57425500 0.02638900 -0.65175600

C -2.27031200 -0.81666600 0.24945000

H -2.88231300 -0.23566900 0.95820700

H -2.93253200 -1.44799300 -0.35136500

H -1.60037300 -1.47012000 0.82724200

G^0 = -308.420155 hartrees

DME *tgg´* (DMSO – SMD, G3MP3B3)

0 1

C -2.49156600 -0.50285800 -0.11040800

H -3.14056800 0.31593300 0.23964300

H -2.56735000 -0.55712900 -1.20806800

H -2.85020400 -1.44464500 0.31573000

O -1.15302100 -0.32797500 0.31667400

C -0.58896500 0.88028100 -0.17199000

H -1.16728200 1.74551600 0.19705900

H -0.62210900 0.90682500 -1.27390000

C 0.85126700 1.02505700 0.29534300

H 0.90465100 0.84936200 1.38193800

H 1.16091600 2.05964400 0.10546900

O 1.78896500 0.20945600 -0.39239100

C 1.83081600 -1.13982900 0.04832800

H 0.91134200 -1.68682400 -0.19206000

H 2.67526300 -1.61344400 -0.46318200

H 1.99847800 -1.20299600 1.13546700

G^0 = -308.419167 hartrees

DME *gtg* (DMSO – SMD, G3MP3B3)

0 1

C -2.38800700 0.75185700 0.19292800

H -1.87122800 1.21958900 1.04325000

H -2.35732800 1.45006300 -0.65829100

H -3.43288400 0.58449800 0.47235200

O -1.84506900 -0.51315600 -0.15308400

C -0.49168700 -0.45465300 -0.58859400

H -0.33256300 0.42118900 -1.23548500

H -0.31827700 -1.35333500 -1.19165500

C 0.49168700 -0.45464900 0.58859600

H 0.31827600 -1.35332600 1.19166400

H 0.33256300 0.42119800 1.23548000

O 1.84506800 -0.51315600 0.15308500

C 2.38800900 0.75185600 -0.19293100

H 2.35733700 1.45006100 0.65828900

H 3.43288300 0.58449300 -0.47236200

H 1.87122500 1.21958900 -1.04324800

G^0 = -308.417413 hartrees

DME *gtg´* (DMSO – SMD, G3MP3B3)

0 1

C -2.64264500 -0.35856900 0.08255600

H -2.33267600 -1.37193500 -0.21005000

H -2.80823100 -0.34713800 1.17128500

H -3.58768400 -0.12817200 -0.41911500

O -1.71290500 0.63895100 -0.31186100

C -0.44732400 0.52954600 0.32841200

H -0.56590100 0.29861300 1.39869900

H 0.01683600 1.51837600 0.24720600

C 0.44732300 -0.52954800 -0.32840300

H 0.56589600 -0.29861800 -1.39869200

H -0.01683400 -1.51837900 -0.24719400

O 1.71290600 -0.63894600 0.31186700

C 2.64264600 0.35856700 -0.08256800

H 2.33267400 1.37193900 0.21001300

H 3.58768300 0.12818300 0.41911400

H 2.80823800 0.34711200 -1.17129500

G^0 = -308.417457 hartrees

DME *ggg* (DMSO – SMD, G3MP3B3)

0 1

C 2.27383700 -0.46634700 -0.37717200

H 1.81226800 -0.69117700 -1.34944200

H 2.86856000 0.45572500 -0.47897700

H 2.94631600 -1.29005300 -0.11722600

O 1.32402000 -0.36387100 0.66914200

C 0.49336500 0.78435700 0.58445400

H 1.10133800 1.70288200 0.50846800

H -0.04358600 0.82554900 1.53799200

C -0.49336500 0.78435400 -0.58445800

H 0.04358600 0.82554000 -1.53799700

H -1.10133900 1.70287800 -0.50847800

O -1.32401900 -0.36387500 -0.66913900

C -2.27383700 -0.46634500 0.37717500

H -2.86856100 0.45572800 0.47897300

H -2.94631600 -1.29005200 0.11723300

H -1.81226900 -0.69116800 1.34944600

G^0 = -308.419023 hartrees

DME *ggg´* (DMSO – SMD, G3MP3B3)

0 1

C -2.04607500 -0.70608000 0.40594800

H -1.44272200 -1.00439100 1.27520400

H -2.79809500 0.02592300 0.74098900

H -2.56229400 -1.59184400 0.02317000

O -1.26227300 -0.19216900 -0.65852000

C -0.60682200 1.03324200 -0.34687200

H -1.31506500 1.73286400 0.12931500

H -0.30380000 1.46159400 -1.30923600

C 0.62835300 0.91240200 0.54316000

H 0.38827900 0.39754200 1.48663700

H 0.94816300 1.93100900 0.79775300

O 1.74386200 0.29774800 -0.08855400

C 1.70520300 -1.12185800 -0.08649500

H 0.88797600 -1.51781100 -0.70130800

H 2.66075600 -1.46593900 -0.49609500

H 1.60013800 -1.51981200 0.93572700

G^0 = -308.417813 hartrees

DME *ttt* (gas phase, B3LYP-GD3BJ/6-311++G**)

0 1

C 2.98301400 -0.11505500 0.00005400

H 3.11533400 -0.74241700 0.89253100

H 3.11540400 -0.74244700 -0.89239100

H 3.73966200 0.66951300 0.00006900

O 1.72046900 0.51977500 -0.00000500

C 0.64461000 -0.39977200 -0.00002700

H 0.68237500 -1.04528700 0.88890100

H 0.68234500 -1.04521200 -0.88901100

C -0.64461000 0.39977200 0.00002500

H -0.68235200 1.04531600 -0.88888300

H -0.68236800 1.04518300 0.88902900

O -1.72046900 -0.51977500 -0.00005300

C -2.98301400 0.11505500 -0.00001400

H -3.11535300 0.74251900 -0.89241700

H -3.73966200 -0.66951300 -0.00010300

H -3.11538500 0.74234600 0.89250600

G^0 = -308.862982 hartrees

DME *tgt* (gas phase, B3LYP-GD3BJ/6-311++G**)

0 1

C 2.73997000 -0.45595300 0.07518000

H 3.33016900 0.45620800 -0.09658900

H 2.75634800 -0.68676800 1.14950100

H 3.19420000 -1.28128900 -0.47318200

O 1.41797300 -0.31467000 -0.40030100

C 0.71869600 0.73951100 0.23254100

H 1.18234500 1.70672700 -0.01848900

H 0.74600000 0.62112900 1.32541800

C -0.71869600 0.73951100 -0.23254100

H -0.74600000 0.62112900 -1.32541800

H -1.18234500 1.70672700 0.01848900

O -1.41797300 -0.31467000 0.40030100

C -2.73997000 -0.45595300 -0.07518000

H -2.75634800 -0.68676800 -1.14950100

H -3.19420000 -1.28128900 0.47318200

H -3.33016900 0.45620800 0.09659000

G^0 = -308.862340 hartrees

DME *ttg* (gas phase, B3LYP-GD3BJ/6-311++G**)

0 1

C 2.82386900 0.38855000 0.04015000

H 2.73121500 1.44409100 -0.25196500

H 3.00156300 0.33830300 1.12332200

H 3.67287900 -0.05290900 -0.48202100

O 1.67534000 -0.34944500 -0.32722500

C 0.49382000 0.12662000 0.28993200

H 0.31332600 1.17388900 0.00557600

H 0.58019000 0.08512900 1.38524600

C -0.65401000 -0.76893100 -0.16479500

H -0.45400000 -1.79476000 0.14971300

H -0.72232900 -0.75465600 -1.26082200

O -1.89017000 -0.39696700 0.42401200

C -2.53586500 0.68911700 -0.21663200

H -2.70533600 0.47574700 -1.28094400

H -3.49876100 0.82077700 0.27741600

H -1.96698500 1.62355700 -0.13174600

G^0 = -308.861118 hartrees

DME *tgg* (gas phase, B3LYP-GD3BJ/6-311++G**)

0 1

C 2.56287300 -0.53435500 -0.01384100

H 3.16287500 0.20548700 0.53550100

H 2.38295300 -1.39655700 0.64414700

H 3.12100300 -0.86581100 -0.88940600

O 1.35027100 0.02234600 -0.47616100

C 0.52682100 0.48838300 0.57168100

H 1.02294000 1.30695600 1.11871700

H 0.33942600 -0.31784500 1.29708900

C -0.77516600 1.02302200 -0.00201800

H -0.54624600 1.76598700 -0.76821100

H -1.33092600 1.51897800 0.81009900

O -1.59470300 0.05971300 -0.63646800

C -2.17544500 -0.88490700 0.23968000

H -2.70728800 -0.39008600 1.06571000

H -2.89021000 -1.46205200 -0.34706300

H -1.43356600 -1.57439300 0.66144300

G^0 = -308.860578 hartrees

DME *tgg'* (gas phase, B3LYP-GD3BJ/6-311++G**)

0 1

C -2.47728800 -0.51876100 -0.11200700

H -3.13437200 0.28252700 0.25460500

H -2.54881200 -0.55707400 -1.20778400

H -2.81180300 -1.47027000 0.30147400

O -1.14262600 -0.32208800 0.31020400

C -0.59243200 0.89846800 -0.15852400

H -1.17018600 1.74872100 0.23948300

H -0.63676800 0.93993500 -1.25661900

C 0.85161400 1.02797200 0.29195600

H 0.91546100 0.84128500 1.37337200

H 1.17534200 2.05539500 0.10250100

O 1.75743800 0.19910200 -0.41286400

C 1.83229000 -1.13992700 0.05897800

H 0.89115100 -1.67605900 -0.08796400

H 2.62848600 -1.62388300 -0.50746800

H 2.08789800 -1.16319700 1.12725900

G^0 = -308.862481 hartrees

DME *gtg* (gas phase, B3LYP-GD3BJ/6-311++G**)

0 1

C -2.35894900 0.77357900 0.18975900

H -1.83009800 1.23324400 1.03419400

H -2.30824100 1.45572800 -0.67025000

H -3.40315900 0.63110100 0.46833100

O -1.84271700 -0.50279700 -0.14132900

C -0.49410200 -0.48681800 -0.58561400

H -0.32071600 0.37576200 -1.24351200

H -0.34641800 -1.39403700 -1.17439100

C 0.49410100 -0.48681400 0.58561600

H 0.34641700 -1.39402900 1.17440000

H 0.32071600 0.37577100 1.24350800

O 1.84271600 -0.50279700 0.14133000

C 2.35895000 0.77357700 -0.18976100

H 2.30825300 1.45572500 0.67025000

H 3.40315700 0.63109500 -0.46834300

H 1.83009400 1.23324600 -1.03419000

G^0 = -308.859083 hartrees

DME *gtg'* (gas phase, B3LYP-GD3BJ/6-311++G**)

0 1

C -2.63781100 -0.35934900 0.08354100

H -2.33228800 -1.36504400 -0.23018900

H -2.77939100 -0.35814800 1.17298000

H -3.58501700 -0.11664800 -0.39845000

O -1.70993900 0.63673900 -0.31034200

C -0.44302300 0.53582700 0.32232700

H -0.55646800 0.31992800 1.39366500

H 0.01838100 1.52020300 0.22251600

C 0.44302200 -0.53582800 -0.32232200

H 0.55646500 -0.31993000 -1.39366100

H -0.01838000 -1.52020500 -0.22250900

O 1.70993900 -0.63673600 0.31034600

C 2.63781100 0.35934800 -0.08354800

H 2.33228600 1.36504700 0.23016700

H 3.58501600 0.11665400 0.39845100

H 2.77939600 0.35813100 -1.17298600

G^0 = -308.859434 hartrees

DME *ggg* (gas phase, B3LYP-GD3BJ/6-311++G**)

0 1

C 2.07586200 -0.59275700 -0.42132900

H 1.47225200 -0.89134800 -1.28541300

H 2.70232700 0.26777400 -0.69723400

H 2.72203200 -1.42359600 -0.13808800

O 1.26967600 -0.30000200 0.70624200

C 0.49698700 0.87740800 0.58313000

H 1.14625900 1.76090400 0.46582100

H -0.02487600 0.97833900 1.53658400

C -0.49698700 0.87741400 -0.58312200

H 0.02487600 0.97835400 -1.53657500

H -1.14625900 1.76090900 -0.46580400

O -1.26967600 -0.29999500 -0.70624500

C -2.07586200 -0.59276100 0.42132300

H -2.70232700 0.26776700 0.69723600

H -2.72203200 -1.42359700 0.13807400

H -1.47225200 -0.89136000 1.28540400

G^0 = -308.859191 hartrees

DME *ggg'* (gas phase, B3LYP-GD3BJ/6-311++G**)

0 1

C -2.05135400 -0.68651100 0.41333300

H -1.45762900 -0.97207000 1.29088100

H -2.79237200 0.06475400 0.72074900

H -2.57270300 -1.56947300 0.04341100

O -1.24914000 -0.20616200 -0.64832300

C -0.59268000 1.02464300 -0.37255300

H -1.30581300 1.73938600 0.06873400

H -0.27098600 1.41276200 -1.34126400

C 0.62588300 0.91703500 0.53990200

H 0.36984000 0.41176900 1.48300700

H 0.94740900 1.93295700 0.78860300

O 1.74461900 0.29310500 -0.06251200

C 1.69020800 -1.12627900 -0.09270500

H 0.88904500 -1.48951700 -0.74203900

H 2.65410200 -1.46694800 -0.47163200

H 1.54292700 -1.53249200 0.91836300

G^0 = -308.860283 hartrees

3*O tt$_n$t* (gas phase, B3LYP-GD3BJ/6-311++G**)

0 1

C 4.71763000 -0.38082600 0.00009600

H 4.77200900 -1.01921100 0.89301100

H 4.77203700 -1.02025000 -0.89207400

H 5.56476800 0.30510000 -0.00029500

O 3.54243800 0.40419900 -0.00037300

C 2.36188500 -0.37596300 0.00004300

H 2.32014400 -1.02131400 0.88917500

H 2.31983900 -1.02181100 -0.88871400

C 1.18134500 0.57657500 -0.00003500

H 1.22209700 1.22101900 -0.88896900

H 1.22222000 1.22133200 0.88866800

O 0.00000000 -0.20429400 0.00018200

C -1.18134500 0.57657500 0.00018700

H -1.22205800 1.22132800 -0.88852400

H -1.22226000 1.22102300 0.88911300

C -2.36188500 -0.37596300 -0.00009400

H -2.32000500 -1.02179600 0.88868100

H -2.31997800 -1.02132900 -0.88920800

O -3.54243800 0.40419900 0.00008900

C -4.71763000 -0.38082600 -0.00012000

H -4.77203300 -1.01995100 0.89226500

H -4.77201300 -1.01951100 -0.89282100

H -5.56476800 0.30510000 0.00003800

G^0 = -462.695814 hartrees

3*O tg$_n$t* (gas phase, B3LYP-GD3BJ/6-311++G**)

0 1

C 3.85984800 1.18598900 0.26896000

H 4.68473500 0.77138400 -0.32896900

H 3.47425800 2.08079800 -0.23931000

H 4.24061000 1.47003500 1.25011700

O 2.83949700 0.23221500 0.47546100

C 2.26587700 -0.22544900 -0.73455200

H 3.01416100 -0.77990800 -1.32266200

H 1.91338900 0.62159700 -1.34023200

C 1.09989800 -1.13481500 -0.42558900

H 1.39541000 -1.85061700 0.35543100

H 0.83343100 -1.70399800 -1.32945500

O 0.00000100 -0.35534500 -0.00000400

C -1.09989500 -1.13480900 0.42559600

H -0.83342500 -1.70398200 1.32946700

H -1.39541200 -1.85062000 -0.35541500

C -2.26587200 -0.22543900 0.73455400

H -1.91338100 0.62161500 1.34022200

H -3.01415300 -0.77989000 1.32267500

O -2.83949900 0.23221000 -0.47546100

C -3.85985400 1.18598200 -0.26896500

H -3.47426700 2.08079500 0.23929900

H -4.68473900 0.77137600 0.32896700

H -4.24061800 1.47001900 -1.25012400

G^0 = -462.695028 hartrees

4O tt$_n$t (gas phase, B3LYP-GD3BJ/6-311++G**)

0 1
C -6.50070600 0.27772400 0.00010300
H -6.57499900 0.91400500 0.89306300
H -6.57505900 0.91514500 -0.89204100
H -7.32590000 -0.43444700 -0.00033100
O -5.30144400 -0.47017900 -0.00040900
C -4.14590900 0.34653500 0.00005700
H -4.12422800 0.99281700 0.88921100
H -4.12392200 0.99340900 -0.88866000
C -2.93639500 -0.56893500 -0.00005400
H -2.95699300 -1.21422700 -0.88907300
H -2.95712000 -1.21462400 0.88867400
O -1.77973300 0.24826000 0.00020900
C -0.57477000 -0.49492500 0.00018300
H -0.51366000 -1.13816900 -0.88858000
H -0.51345500 -1.13791300 0.88911800
C 0.57477000 0.49492500 -0.00007800
H 0.51357300 1.13824500 0.88862400
H 0.51354200 1.13783700 -0.88907300
O 1.77973300 -0.24826000 0.00006800
C 2.93639500 0.56893500 -0.00010400
H 2.95707100 1.21459000 0.88865000
H 2.95704200 1.21426100 -0.88909700
C 4.14590900 -0.34653500 0.00004500
H 4.12408500 -0.99295400 0.88909700
H 4.12406500 -0.99327200 -0.88877400
O 5.30144400 0.47017900 -0.00011400
C 6.50070600 -0.27772400 0.00001700
H 6.57502300 -0.91474200 -0.89241600
H 6.57503500 -0.91440800 0.89268800
H 7.32590000 0.43444700 -0.00012200
G^0 = -616.528648 hartrees

4O tg$_n$t (gas phase, B3LYP-GD3BJ/6-311++G**)

0 1
C -5.10758700 -1.19633600 0.86860800
H -5.78838100 -1.61783300 0.11445500
H -4.53326200 -2.01758600 1.31974600
H -5.69657000 -0.70592400 1.64380700
O -4.25539700 -0.21973900 0.30892800
C -3.43781800 -0.73517100 -0.72474700
H -4.05937900 -1.05056500 -1.57766100
H -2.87269300 -1.60941500 -0.37109600
C -2.47002400 0.33157000 -1.18002800
H -3.01339000 1.27721000 -1.32219200
H -2.03447700 0.03779100 -2.14750700
O -1.45009000 0.48086800 -0.21283300
C -0.56261400 1.54296700 -0.50378700
H -1.09502400 2.50519000 -0.45425700
H -0.14326600 1.43392500 -1.51442400
C 0.56261100 1.54296600 0.50379100
H 0.14326400 1.43391700 1.51442800
H 1.09501800 2.50519100 0.45426700
O 1.45009000 0.48087000 0.21283200
C 2.47002300 0.33156800 1.18002700
H 2.03447500 0.03778700 2.14750500
H 3.01339000 1.27720800 1.32219400
C 3.43781600 -0.73517200 0.72474300
H 4.05937700 -1.05056900 1.57765800
H 2.87269100 -1.60941500 0.37109100
O 4.25539600 -0.21973900 -0.30893000
C 5.10759300 -1.19633400 -0.86860500
H 4.53327200 -2.01758700 -1.31974200
H 5.78838600 -1.61782500 -0.11445000
H 5.69657500 -0.70592100 -1.64380500
G^0 = -616.527746 hartrees

5*O* *tt$_n$t* (gas phase, B3LYP-GD3BJ/6-311++G**)

0 1
C 8.26026400 -0.42023400 0.00013300
H 8.31455200 -1.05865600 0.89286700
H 8.31456100 -1.05919900 -0.89221300
H 9.10723500 0.26579100 -0.00007300
O 7.08477800 0.36464400 -0.00011100
C 5.90436600 -0.41549000 0.00012300
H 5.86228200 -1.06059700 0.88924900
H 5.86218800 -1.06099300 -0.88871100
C 4.72392900 0.53754700 -0.00003300
H 4.76480400 1.18164600 -0.88904400
H 4.76481600 1.18195900 0.88875100
O 3.54216600 -0.24289800 0.00011200
C 2.36117100 0.53754900 0.00002000
H 2.31992500 1.18193700 -0.88888800
H 2.31983800 1.18202000 0.88886400
C 1.18135200 -0.41633900 0.00001300
H 1.22249800 -1.06071300 0.88895600
H 1.22250700 -1.06071900 -0.88892600
O 0.00000000 0.36383900 0.00000300
C -1.18135200 -0.41633900 -0.00000600
H -1.22250900 -1.06071500 0.88893500
H -1.22249600 -1.06071600 -0.88894600
C -2.36117100 0.53754900 -0.00001600
H -2.31989000 1.18197700 0.88886200
H -2.31987300 1.18198000 -0.88889000
O -3.54216600 -0.24289800 -0.00002900
C -4.72392900 0.53754700 -0.00002700
H -4.76479500 1.18182400 -0.88890900
H -4.76482500 1.18178000 0.88888600
C -5.90436600 -0.41549000 -0.00006400
H -5.86221000 -1.06085600 0.88887000
H -5.86226000 -1.06073400 -0.88909000
O -7.08477800 0.36464400 0.00002100
C -8.26026400 -0.42023400 -0.00014000
H -8.31453200 -1.05878000 -0.89278700
H -8.31458100 -1.05907500 0.89229400
H -9.10723500 0.26579100 -0.00004800
G^0 = -770.361785 hartrees

5*O* *tg*$_n$*t* (gas phase, B3LYP-GD3BJ/6-311++G**)

0 1
C -6.62512200 -1.11712400 -0.31515600
H -7.21644600 -0.95482700 -1.22834000
H -6.13148200 -2.09638400 -0.38715200
H -7.29423200 -1.11810900 0.54545100
O -5.68650300 -0.08164700 -0.11503400
C -4.75892200 0.02837100 -1.17804400
H -5.27643400 0.32903500 -2.10276500
H -4.26404100 -0.93623500 -1.36075300
C -3.71574900 1.06418700 -0.83023600
H -4.21353300 1.96070900 -0.43247900
H -3.17068700 1.35135200 -1.74271100
O -2.82077900 0.52468300 0.12144500
C -1.87697200 1.46772900 0.59075700
H -2.38690100 2.26701300 1.15016300
H -1.33604300 1.92753600 -0.24905800
C -0.88858500 0.77531500 1.49916400
H -1.43524200 0.14109100 2.21196800
H -0.32893800 1.53140400 2.07097000
O 0.00000000 -0.00000500 0.71866000
C 0.88858500 -0.77533600 1.49915400
H 0.32893800 -1.53143200 2.07095000
H 1.43524200 -0.14112100 2.21196700
C 1.87697300 -1.46773700 0.59073800
H 2.38690200 -2.26702800 1.15013400
H 1.33604400 -1.92753400 -0.24908300
O 2.82078000 -0.52468500 0.12143900
C 3.71574900 -1.06417700 -0.83025000
H 3.17068700 -1.35133000 -1.74272700
H 4.21353500 -1.96070300 -0.43250400
C 4.75892200 -0.02835500 -1.17804400
H 4.26404000 0.93625200 -1.36074100
H 5.27643400 -0.32900800 -2.10276900
O 5.68650300 0.08165000 -0.11503300
C 6.62512100 1.11713000 -0.31514200
H 7.21644500 0.95484500 -1.22832800
H 6.13148000 2.09639100 -0.38712600
H 7.29423100 1.11810500 0.54546400
G^0 = -770.360178 hartrees

6*O* tt_nt (gas phase, B3LYP-GD3BJ/6-311++G**)

0 1
C -10.03693800 0.34006800 0.00014900
H -10.10032500 0.97727400 0.89333600
H -10.10035200 0.97892500 -0.89185800
H -10.87419700 -0.35792200 -0.00049100
O -8.85067200 -0.42831200 -0.00057300
C -7.68126600 0.36832500 0.00013100
H -7.64838900 1.01392900 0.88949500
H -7.64803700 1.01499500 -0.88844600
C -6.48785800 -0.56804600 -0.00020800
H -6.51956900 -1.21264200 -0.88943100
H -6.51973100 -1.21350500 0.88838500
O -5.31711300 0.22902100 0.00028300
C -4.12524100 -0.53483900 0.00006800
H -4.07502700 -1.17882800 -0.88887700
H -4.07482200 -1.17902400 0.88886000
C -2.95910400 0.43541600 0.00005500
H -3.00919800 1.07939700 0.88899400
H -3.00922500 1.07943300 -0.88885600
O -1.76670200 -0.32817500 0.00001700
C -0.59639600 0.46865200 0.00006200
H -0.56419900 1.11375200 0.88901900
H -0.56421800 1.11388500 -0.88879900
C 0.59639600 -0.46865200 -0.00002200
H 0.56421300 -1.11389200 0.88883400
H 0.56420400 -1.11374500 -0.88898400
O 1.76670200 0.32817500 0.00004000
C 2.95910400 -0.43541600 -0.00002000
H 3.00920000 -1.07935600 -0.88898800
H 3.00922200 -1.07947400 0.88886300
C 4.12524100 0.53483900 0.00004600
H 4.07481400 1.17898700 0.88886400
H 4.07503500 1.17886600 -0.88887200
O 5.31711300 -0.22902100 0.00024300
C 6.48785800 0.56804500 -0.00033900
H 6.51962800 1.21245100 -0.88969800
H 6.51967300 1.21369600 0.88811800
C 7.68126600 -0.36832500 0.00028700
H 7.64830100 -1.01376500 0.88976700
H 7.64812500 -1.01515900 -0.88817400
O 8.85067200 0.42831200 -0.00045100
C 10.03693800 -0.34006900 0.00009000
H 10.10026800 -0.97883700 -0.89198600
H 10.10040900 -0.97736400 0.89320900
H 10.87419700 0.35792200 -0.00055300
G^0 = -924.194193 hartrees

6*O* *tg*$_n$*t* (gas phase, B3LYP-GD3BJ/6-311++G**)

0 1
C -8.07781300 -0.64833900 0.84281700
H -8.71963400 -1.25739000 0.18935200
H -7.60304500 -1.30906200 1.58161800
H -8.69497000 0.08345600 1.36428100
O -7.11464300 0.06874100 0.10030500
C -6.24985300 -0.77691000 -0.63419700
H -6.81664700 -1.31803500 -1.40829600
H -5.78030600 -1.51861700 0.02770600
C -5.17353000 0.05376900 -1.29260800
H -5.63439400 0.92908600 -1.77340400
H -4.67900000 -0.54505300 -2.07297200
O -4.23190000 0.45574400 -0.31821800
C -3.24990100 1.33685000 -0.82767000
H -3.71552400 2.28668800 -1.13236900
H -2.75372100 0.90003400 -1.70646400
C -2.21837400 1.60732800 0.24206900
H -2.72987500 1.83816700 1.18772100
H -1.61713500 2.48335500 -0.04609900
O -1.38648200 0.47305500 0.38677400
C -0.46248600 0.59709000 1.45007900
H -0.99618100 0.64352000 2.41177200
H 0.13254400 1.51567100 1.34307600
C 0.46248600 -0.59706100 1.45009100
H 0.99618100 -0.64347200 2.41178500
H -0.13254400 -1.51564400 1.34310600
O 1.38648200 -0.47304800 0.38678300
C 2.21837400 -1.60732300 0.24210100
H 1.61713600 -2.48335600 -0.04604900
H 2.72987600 -1.83814300 1.18775800
C 3.24990100 -1.33686700 -0.82764300
H 2.75372000 -0.90006900 -1.70644600
H 3.71552400 -2.28671100 -1.13232300
O 4.23190000 -0.45575100 -0.31820900
C 5.17352900 -0.05379500 -1.29260700
H 5.63439400 -0.92912200 -1.77338500
H 4.67900000 0.54501100 -2.07298400
C 6.24985300 0.77689700 -0.63421400
H 6.81664700 1.31800700 -1.40832400
H 5.78030600 1.51861800 0.02767400
O 7.11464300 -0.06873900 0.10030600
C 8.07781300 0.64835600 0.84280300
H 7.60304500 1.30909500 1.58159000
H 8.71963400 1.25739400 0.18932500
H 8.69497000 -0.08342800 1.36428200
G^0 = -924.192971 hartrees

7*O* tt_nt (gas phase, B3LYP-GD3BJ/6-311++G**)

0 1
C 11.80247500 -0.43695900 0.00013300
H 11.85701900 -1.07481000 0.89347800
H 11.85699600 -1.07685100 -0.89175500
H 12.64938500 0.24931800 -0.00066900
O 10.62702600 0.34784600 -0.00074300
C 9.44663800 -0.43238800 0.00014700
H 9.40475000 -1.07730200 0.88967100
H 9.40435700 -1.07874800 -0.88830900
C 8.26639600 0.52055400 -0.00038100
H 8.30710300 1.16447400 -0.88975500
H 8.30729700 1.16571200 0.88808800
O 7.08462500 -0.26008600 0.00028900
C 5.90355000 0.52034000 -0.00007200
H 5.86228800 1.16480600 -0.88916000
H 5.86210900 1.16537300 0.88859700
C 4.72396800 -0.43356100 0.00012900
H 4.76509300 -1.07801500 0.88921900
H 4.76509000 -1.07841100 -0.88867400
O 3.54231900 0.34657600 -0.00004800
C 2.36100700 -0.43373900 0.00017400
H 2.31973800 -1.07817700 0.88928100
H 2.31972200 -1.07866400 -0.88857900
C 1.18149100 0.52025500 -0.00007700
H 1.22272000 1.16514800 0.88866400
H 1.22269600 1.16465200 -0.88917900
O 0.00000000 -0.26004800 0.00015900
C -1.18149100 0.52025500 -0.00002700
H -1.22271800 1.16466600 -0.88911700
H -1.22269900 1.16513400 0.88872500
C -2.36100700 -0.43374000 0.00025400
H -2.31959500 -1.07831400 0.88925600
H -2.31986500 -1.07852800 -0.88860400
O -3.54231900 0.34657600 0.00033700
C -4.72396800 -0.43356100 -0.00006800
H -4.76509200 -1.07774500 -0.88935400
H -4.76509100 -1.07868100 0.88853900
C -5.90355000 0.52033900 0.00043500
H -5.86226700 1.16454400 0.88971200
H -5.86213100 1.16563400 -0.88804500
O -7.08462500 -0.26008600 -0.00013000
C -8.26639600 0.52055400 0.00031900
H -8.30714600 1.16568500 -0.88817600
H -8.30725500 1.16450200 0.88966700
C -9.44663800 -0.43238800 -0.00038500
H -9.40450900 -1.07741800 -0.88981500
H -9.40459900 -1.07863200 0.88816500
O -10.62702600 0.34784600 0.00009700
C -11.80247500 -0.43695900 -0.00051200
H -12.64938500 0.24931800 0.00025100
H -11.85711500 -1.07497700 -0.89373400
H -11.85690000 -1.07668500 0.89150000
G^0 = -1078.026849 hartrees

7*O* *tg*$_n$*t* (gas phase, B3LYP-GD3BJ/6-311++G**)

0 1
C 9.40606200 -1.38035200 -0.31243700
H 10.04514400 -1.51657100 0.57238500
H 8.86776300 -2.31927900 -0.50336600
H 10.03500000 -1.14933800 -1.17232300
O 8.51242600 -0.30035300 -0.14456000
C 7.64029100 -0.47849200 0.95551400
H 8.21333700 -0.48353400 1.89621000
H 7.10656200 -1.43625700 0.87459100
C 6.63552800 0.64900900 0.98925400
H 7.15886900 1.60574200 0.84595000
H 6.14683100 0.67017400 1.97558600
O 5.67309200 0.44788700 -0.02608400
C 4.75404000 1.51705600 -0.13719700
H 5.27433600 2.43106600 -0.46236300
H 4.27527200 1.72053200 0.83165300
C 3.69259200 1.15993200 -1.15075600
H 4.17456800 0.75323400 -2.05159100
H 3.14244100 2.06970400 -1.43642100
O 2.80693600 0.21311900 -0.58628200
C 1.84554400 -0.26245200 -1.50779000
H 2.34043800 -0.82423700 -2.31487500
H 1.29352500 0.57405300 -1.96040600
C 0.87335600 -1.17042600 -0.79225900
H 0.29885800 -1.74484800 -1.53521100
H 1.43426300 -1.88117000 -0.16809200
O 0.00000000 -0.39001800 -0.00000400
C -0.87335600 -1.17043200 0.79224700
H -0.29885700 -1.74485800 1.53519400
H -1.43426200 -1.88117100 0.16807400
C -1.84554300 -0.26246200 1.50778400
H -1.29352300 0.57404000 1.96040500
H -2.34043700 -0.82425200 2.31486600
O -2.80693500 0.21311500 0.58628000
C -3.69259000 1.15992500 1.15076000
H -4.17456500 0.75322200 2.05159400
H -3.14243900 2.06969500 1.43643000
C -4.75404000 1.51705500 0.13720500
H -5.27433600 2.43106300 0.46237700
H -4.27527300 1.72053600 -0.83164400
O -5.67309200 0.44788600 0.02608800
C -6.63552900 0.64901500 -0.98924800
H -6.14683400 0.67018400 -1.97558100
H -7.15887000 1.60574700 -0.84593800
C -7.64029300 -0.47848700 -0.95551300
H -8.21334000 -0.48352400 -1.89620800
H -7.10656400 -1.43625200 -0.87459500
O -8.51242600 -0.30035300 0.14456400
C -9.40606300 -1.38035200 0.31243700
H -10.03499900 -1.14934300 1.17232400
H -10.04514600 -1.51656700 -0.57238500
H -8.86776300 -2.31928100 0.50335900
G^0 = -1078.025561 hartrees

7*O* tt_nt – K^+ (gas phase, B3LYP-GD3BJ/6-311++G**)

1 1 0 1 1 1
C(Fragment=1) -11.82000900 0.09127900 0.00008300
H(Fragment=1) -11.88405900 0.72699400 0.89332800
H(Fragment=1) -11.88406000 0.72723300 -0.89299100
H(Fragment=1) -12.65297900 -0.61069600 -0.00001000
O(Fragment=1) -10.62867900 -0.67490300 -0.00002000
C(Fragment=1) -9.46212900 0.11902900 0.00008700
H(Fragment=1) -9.42399900 0.76469200 0.88949700
H(Fragment=1) -9.42398700 0.76491300 -0.88916200
C(Fragment=1) -8.27844000 -0.83001200 -0.00002300
H(Fragment=1) -8.31442000 -1.47191000 -0.88943800
H(Fragment=1) -8.31442200 -1.47211600 0.88924200
O(Fragment=1) -7.09005200 -0.04664200 0.00006800
C(Fragment=1) -5.91493900 -0.82521200 0.00000300
H(Fragment=1) -5.86412200 -1.46884200 -0.88903800
H(Fragment=1) -5.86407700 -1.46892500 0.88898200
C(Fragment=1) -4.75087700 0.14799300 0.00002100
H(Fragment=1) -4.79804400 0.78677100 0.89153800
H(Fragment=1) -4.79806400 0.78683800 -0.89144800
O(Fragment=1) -3.54221500 -0.61009400 -0.00002500
C(Fragment=1) -2.38874000 0.17599000 0.00003900
H(Fragment=1) -2.35775000 0.82267000 0.89709200
H(Fragment=1) -2.35786900 0.82296900 -0.89680600
C(Fragment=1) -1.19376800 -0.76108600 -0.00018600
H(Fragment=1) -1.21566200 -1.39714000 0.88958600
H(Fragment=1) -1.21567300 -1.39675500 -0.89023300
O(Fragment=1) -0.00000300 0.03816500 -0.00001900
C(Fragment=1) 1.19376200 -0.76108500 -0.00018900
H(Fragment=1) 1.21566900 -1.39675000 -0.89023900
H(Fragment=1) 1.21565400 -1.39714400 0.88958000
C(Fragment=1) 2.38873400 0.17599000 0.00004500
H(Fragment=1) 2.35773100 0.82267800 0.89709200
H(Fragment=1) 2.35787700 0.82296100 -0.89680600
O(Fragment=1) 3.54220900 -0.61009300 0.00000500
C(Fragment=1) 4.75087200 0.14799400 0.00001000
H(Fragment=1) 4.79805800 0.78678900 -0.89149400
H(Fragment=1) 4.79804100 0.78682000 0.89149200
C(Fragment=1) 5.91493400 -0.82521200 0.00004400
H(Fragment=1) 5.86408100 -1.46886600 0.88906600
H(Fragment=1) 5.86410700 -1.46890000 -0.88895400
O(Fragment=1) 7.09004800 -0.04664300 0.00004500
C(Fragment=1) 8.27843400 -0.83001300 0.00003000
H(Fragment=1) 8.31441400 -1.47200000 -0.88932000
H(Fragment=1) 8.31441700 -1.47202900 0.88936000
C(Fragment=1) 9.46212500 0.11902600 0.00004600
H(Fragment=1) 9.42399200 0.76481300 -0.88927500
H(Fragment=1) 9.42398700 0.76478700 0.88938500
O(Fragment=1) 10.62867400 -0.67490700 0.00003700
C(Fragment=1) 11.82000400 0.09127300 0.00003300
H(Fragment=1) 12.65297400 -0.61070300 0.00003400
H(Fragment=1) 11.88405500 0.72710400 -0.89312900
H(Fragment=1) 11.88405800 0.72711100 0.89318900
K(Fragment=2) 0.00002300 2.61851900 -0.00005800
G^0 = -1677.827470 hartrees, H^0 = -1677.724687 hartrees

7*O* tg_nt – K^+ (gas phase, B3LYP-GD3BJ/6-311++G**)

1 1 0 1 1 1
C(Fragment=1) 0.29152900 0.45704100 3.46256400
H(Fragment=1) -0.14186300 1.04576600 4.27919600
H(Fragment=1) 0.72101600 -0.46298600 3.87700800
H(Fragment=1) 1.06926300 1.03553600 2.96623900
O(Fragment=1) -0.69117200 0.16075400 2.47343400
C(Fragment=1) -1.77982200 -0.60409900 2.97679900
H(Fragment=1) -2.25074000 -0.08388000 3.82146900
H(Fragment=1) -1.42520700 -1.58211300 3.32787900
C(Fragment=1) -2.80275100 -0.78571600 1.88091700
H(Fragment=1) -3.10828700 0.19660600 1.49967900
H(Fragment=1) -3.68679700 -1.28844800 2.29338000
O(Fragment=1) -2.23320400 -1.57829100 0.84148300
C(Fragment=1) -3.12809400 -1.88071800 -0.22797600
H(Fragment=1) -4.12293900 -2.12510500 0.16343500
H(Fragment=1) -2.72349100 -2.76880200 -0.71664800
C(Fragment=1) -3.24029300 -0.75746200 -1.24286500
H(Fragment=1) -3.56862000 0.17055100 -0.76027400
H(Fragment=1) -3.98589600 -1.02723300 -2.00262200
O(Fragment=1) -1.96167000 -0.58823700 -1.84641500
C(Fragment=1) -1.87004700 0.46681500 -2.79770200
H(Fragment=1) -2.68405500 0.40279500 -3.53089100
H(Fragment=1) -0.92682900 0.29918400 -3.32097000
C(Fragment=1) -1.86390800 1.85241200 -2.16796200
H(Fragment=1) -1.62046900 2.58470900 -2.94886800
H(Fragment=1) -2.84360900 2.12167700 -1.75302900
O(Fragment=1) -0.88772500 1.86980800 -1.13307900
C(Fragment=1) -0.55377400 3.17213800 -0.66658800
H(Fragment=1) -1.45883900 3.77053100 -0.49940000
H(Fragment=1) 0.05059800 3.68939700 -1.42242100
C(Fragment=1) 0.17765900 3.04477300 0.66275700
H(Fragment=1) -0.50982800 2.67684600 1.42542200
H(Fragment=1) 0.54128300 4.03677800 0.96251700
O(Fragment=1) 1.24330200 2.10151600 0.65626400
C(Fragment=1) 2.30553800 2.37656400 -0.24862700
H(Fragment=1) 1.95073400 2.31209100 -1.28438600
H(Fragment=1) 2.71015100 3.38335300 -0.07926800
C(Fragment=1) 3.39642400 1.35146500 -0.02564600
H(Fragment=1) 4.18780300 1.51819800 -0.76635100
H(Fragment=1) 3.82882700 1.45400200 0.97681900
O(Fragment=1) 2.83526900 0.04986400 -0.17243400
C(Fragment=1) 3.76857700 -0.96478300 -0.53013400
H(Fragment=1) 4.69098400 -0.86713900 0.05590600
H(Fragment=1) 4.01868300 -0.87519300 -1.59430100
C(Fragment=1) 3.15963400 -2.31850800 -0.24084500
H(Fragment=1) 3.86005200 -3.09651800 -0.57135800
H(Fragment=1) 2.99969400 -2.44107200 0.83993200
O(Fragment=1) 1.92121500 -2.44058300 -0.93054700
C(Fragment=1) 1.40649400 -3.76860900 -0.91212900
H(Fragment=1) 0.46641000 -3.75494600 -1.46308000
H(Fragment=1) 2.09819900 -4.46366400 -1.40048100
H(Fragment=1) 1.22255900 -4.10927600 0.11451700
K(Fragment=2) 0.17000500 -0.43148200 -0.05968700
G^0 = -1677.911438 hartrees, H^0 = -1677.822628 hartrees

7*O tg$_n$t* – Li^+ (gas phase, B3LYP-GD3BJ/6-311++G**)

1 1 0 1 1 1
C(Fragment=1) 0.11258600 0.89294800 3.16546700
H(Fragment=1) 0.02456200 0.32260800 4.09711800
H(Fragment=1) 0.74696200 1.76787100 3.34419600
H(Fragment=1) -0.87423300 1.20908000 2.83653000
O(Fragment=1) 0.65555600 0.08374200 2.12404900
C(Fragment=1) 1.95675500 -0.40985100 2.43097100
H(Fragment=1) 1.90743200 -1.15016700 3.23923200
H(Fragment=1) 2.59874400 0.41810800 2.75626500
C(Fragment=1) 2.51929400 -1.03205800 1.17380100
H(Fragment=1) 1.91370100 -1.89711100 0.87960500
H(Fragment=1) 3.55161400 -1.36126400 1.34508600
O(Fragment=1) 2.46257400 -0.01050600 0.18590400
C(Fragment=1) 3.03077000 -0.30811100 -1.08154500
H(Fragment=1) 4.05241100 -0.69356400 -0.97569300
H(Fragment=1) 3.07063600 0.64731900 -1.60612600
C(Fragment=1) 2.18536900 -1.29354500 -1.86424100
H(Fragment=1) 2.20810300 -2.28204400 -1.39331000
H(Fragment=1) 2.57790800 -1.39715500 -2.88314000
O(Fragment=1) 0.85321800 -0.78403100 -1.89364200
C(Fragment=1) -0.12404000 -1.72661800 -2.33358400
H(Fragment=1) 0.20842800 -2.24214300 -3.24228800
H(Fragment=1) -1.00997700 -1.13454600 -2.56082800
C(Fragment=1) -0.45053000 -2.72912300 -1.23738300
H(Fragment=1) -1.33614700 -3.30183300 -1.53159900
H(Fragment=1) 0.36166500 -3.44327100 -1.06521900
O(Fragment=1) -0.69375000 -1.99335100 -0.03800700
C(Fragment=1) -1.82039200 -2.42753000 0.71650400
H(Fragment=1) -1.61502200 -3.37512600 1.23009200
H(Fragment=1) -2.66771100 -2.59255300 0.04087800
C(Fragment=1) -2.13122300 -1.35197300 1.74696100
H(Fragment=1) -1.35898900 -1.33956800 2.51329200
H(Fragment=1) -3.09674700 -1.56402000 2.22213400
O(Fragment=1) -2.11130300 -0.04713600 1.17740500
C(Fragment=1) -3.18098600 0.24678400 0.28326800
H(Fragment=1) -3.40142400 -0.60922700 -0.36164400
H(Fragment=1) -4.08830900 0.50664100 0.84161800
C(Fragment=1) -2.73556700 1.40884900 -0.56881300
H(Fragment=1) -3.53764700 1.71089400 -1.25267200
H(Fragment=1) -2.48238100 2.25917300 0.07338900
O(Fragment=1) -1.59331700 0.97289400 -1.30596800
C(Fragment=1) -0.96177800 1.98788300 -2.08713800
H(Fragment=1) -1.69497600 2.52230000 -2.70201200
H(Fragment=1) -0.27011700 1.45343400 -2.73821800
C(Fragment=1) -0.19332200 2.96035600 -1.21004200
H(Fragment=1) 0.44537200 3.59225600 -1.83987700
H(Fragment=1) -0.85902800 3.62170000 -0.64056000
O(Fragment=1) 0.59371800 2.18190900 -0.31811700
C(Fragment=1) 1.46690400 2.96637400 0.48975500
H(Fragment=1) 2.06824700 2.26983600 1.06587000
H(Fragment=1) 2.12388700 3.57911600 -0.13663600
H(Fragment=1) 0.89463100 3.62030400 1.15759700
Li(Fragment=2) 0.05392900 0.18282200 0.03001700
G^0 = -1085.493161 hartrees, H^0 = -1085.410897 hartrees

7*O* tg_nt – Na^+ (gas phase, B3LYP-GD3BJ/6-311++G**)

1 1 0 1 1 1
C(Fragment=1) -1.31573800 1.16727500 2.83783300
H(Fragment=1) -1.40123300 0.88516900 3.89311700
H(Fragment=1) -1.31945100 2.26078000 2.75868000
H(Fragment=1) -2.14673900 0.74741500 2.27838500
O(Fragment=1) -0.12581300 0.62414800 2.26790900
C(Fragment=1) 1.05087100 1.04807300 2.94529300
H(Fragment=1) 0.99871800 0.77350400 4.00674800
H(Fragment=1) 1.15559900 2.13843200 2.87319500
C(Fragment=1) 2.24035900 0.37205700 2.31049100
H(Fragment=1) 2.12522700 -0.71817900 2.36813300
H(Fragment=1) 3.15216600 0.65442500 2.85092200
O(Fragment=1) 2.31746400 0.80146000 0.95332100
C(Fragment=1) 3.42721500 0.27377600 0.22780400
H(Fragment=1) 4.28583100 0.11768800 0.89019800
H(Fragment=1) 3.69838300 1.02727900 -0.51241500
C(Fragment=1) 3.06811800 -1.02078900 -0.47462700
H(Fragment=1) 2.77689100 -1.78880700 0.25220800
H(Fragment=1) 3.93386200 -1.39135500 -1.03878500
O(Fragment=1) 1.98779200 -0.72617700 -1.35589400
C(Fragment=1) 1.53838400 -1.81294200 -2.15687600
H(Fragment=1) 2.36908800 -2.25118400 -2.72406200
H(Fragment=1) 0.83439100 -1.36993300 -2.86326000
C(Fragment=1) 0.84516000 -2.89735800 -1.34526800
H(Fragment=1) 0.32699200 -3.57488900 -2.03524700
H(Fragment=1) 1.55461800 -3.49479400 -0.75971300
O(Fragment=1) -0.08121200 -2.26396700 -0.46777400
C(Fragment=1) -1.05359500 -3.15035500 0.07813400
H(Fragment=1) -0.57259500 -4.02723100 0.52964800
H(Fragment=1) -1.71080900 -3.50701700 -0.72451400
C(Fragment=1) -1.82587500 -2.40650600 1.15865300
H(Fragment=1) -1.19030700 -2.24874600 2.03117200
H(Fragment=1) -2.69185100 -3.00979600 1.46127700
O(Fragment=1) -2.22409000 -1.10151900 0.76555700
C(Fragment=1) -3.11533100 -1.02331200 -0.33875300
H(Fragment=1) -2.66186900 -1.46957000 -1.23138100
H(Fragment=1) -4.05715700 -1.54439200 -0.12312100
C(Fragment=1) -3.37951400 0.44509400 -0.59283200
H(Fragment=1) -3.97303400 0.55080500 -1.50855900
H(Fragment=1) -3.93611600 0.89479600 0.23815300
O(Fragment=1) -2.11895400 1.09269200 -0.73119300
C(Fragment=1) -2.15349300 2.32677400 -1.43993300
H(Fragment=1) -2.93810500 2.98289300 -1.04307400
H(Fragment=1) -2.35634800 2.13491600 -2.50048300
C(Fragment=1) -0.80773500 2.99574600 -1.26992000
H(Fragment=1) -0.76782800 3.88988500 -1.90445800
H(Fragment=1) -0.66126600 3.30898100 -0.22639800
O(Fragment=1) 0.20127800 2.06696100 -1.64413000
C(Fragment=1) 1.48781800 2.66221700 -1.79021100
H(Fragment=1) 2.15503100 1.87264500 -2.12898200
H(Fragment=1) 1.46061800 3.46463000 -2.53524300
H(Fragment=1) 1.84630900 3.06168200 -0.83460300
Na(Fragment=2) 0.00176200 0.16388200 -0.08533000
G^0 = -1240.267261 hartrees, H^0 = -1240.181681 hartrees

DME *ttt* (DMSO – SMD, B3LYP-GD3BJ/6-311++G**)

0 1

C 2.98662400 -0.11939400 0.00004600

H 3.11236600 -0.74703800 0.89238000

H 3.11241200 -0.74706300 -0.89226400

H 3.75267100 0.65734400 0.00005400

O 1.72395300 0.52933000 0.00000500

C 0.64685500 -0.39526200 -0.00002100

H 0.69440600 -1.03930100 0.88928500

H 0.69438100 -1.03922300 -0.88938500

C -0.64685500 0.39526200 0.00002800

H -0.69438500 1.03932900 -0.88925900

H -0.69440200 1.03919600 0.88941100

O -1.72395300 -0.52933000 -0.00005100

C -2.98662400 0.11939400 -0.00002200

H -3.11236700 0.74713200 -0.89229000

H -3.75267100 -0.65734400 -0.00011200

H -3.11241100 0.74696900 0.89235500

G^0 = -308.868297 hartrees

DME *tgt* (DMSO – SMD, B3LYP-GD3BJ/6-311++G**)

0 1

C 2.77564800 -0.41098500 0.06451600

H 3.31591400 0.51110300 -0.19160600

H 2.84238400 -0.56540900 1.15018400

H 3.24926200 -1.25258100 -0.44350400

O 1.42608700 -0.35771200 -0.36806600

C 0.71786500 0.71956300 0.23013700

H 1.17833900 1.67677100 -0.05576500

H 0.75891700 0.64035000 1.32598400

C -0.71786500 0.71956300 -0.23013700

H -0.75891700 0.64035000 -1.32598400

H -1.17833900 1.67677100 0.05576500

O -1.42608700 -0.35771200 0.36806600

C -2.77564800 -0.41098600 -0.06451700

H -2.84238400 -0.56540900 -1.15018400

H -3.24926200 -1.25258100 0.44350400

H -3.31591400 0.51110300 0.19160500

G^0 = -308.869182 hartrees

DME *ttg* (DMSO – SMD, B3LYP-GD3BJ/6-311++G**)

0 1

C 2.81024200 0.40947200 0.03822900

H 2.70425100 1.42692300 -0.36152800

H 2.95834600 0.47230100 1.12466400

H 3.68449700 -0.06160100 -0.41366500

O 1.68249000 -0.39061800 -0.28281500

C 0.47872600 0.13497700 0.25865500

H 0.31811500 1.15699700 -0.10933900

H 0.53463600 0.17289500 1.35608300

C -0.65683000 -0.78787600 -0.16645800

H -0.44570700 -1.80125700 0.18286200

H -0.73043900 -0.80893700 -1.26226300

O -1.90593400 -0.41978300 0.40539000

C -2.50076600 0.72197900 -0.20213400

H -2.57585400 0.59451000 -1.29008900

H -3.50477600 0.81809100 0.21428200

H -1.94374500 1.64197100 0.00863400

G^0 = -308.867127 hartrees

DME *tgg* (DMSO – SMD, B3LYP-GD3BJ/6-311++G**)

0 1

C 2.57359600 -0.52825400 -0.00929900

H 3.15635200 0.21550300 0.55174800

H 2.39255900 -1.39429500 0.64202700

H 3.15150900 -0.85097600 -0.87685300

O 1.35683900 0.02069800 -0.48839800

C 0.52539500 0.47637200 0.56886700

H 1.02260700 1.28820700 1.12162700

H 0.34199400 -0.33851900 1.28252500

C -0.77416200 1.02228100 0.01093500

H -0.55364100 1.78622900 -0.73837900

H -1.32504600 1.49769400 0.83591600

O -1.60497100 0.06854800 -0.64181200

C -2.18330800 -0.88521100 0.23759700

H -2.70014100 -0.39327600 1.07306000

H -2.91055200 -1.45514500 -0.34313100

H -1.43971400 -1.58051000 0.64454200

G^0 = -308.868087 hartrees

DME *tgg'* (DMSO – SMD, B3LYP-GD3BJ/6-311++G**)

0 1

C -2.49316000 -0.50484900 -0.11276100

H -3.13465400 0.31114300 0.24752800

H -2.56146500 -0.54669600 -1.20826000

H -2.85105300 -1.44825800 0.30239100

O -1.15126800 -0.33295100 0.31478600

C -0.58908800 0.88304100 -0.16463900

H -1.16535600 1.73854300 0.22020500

H -0.63360200 0.91389900 -1.26297500

C 0.84802100 1.02821200 0.29316500

H 0.91514600 0.84818800 1.37506500

H 1.15552100 2.05970100 0.10096400

O 1.78256400 0.20852300 -0.40123100

C 1.83722200 -1.13982900 0.05329700

H 0.91676600 -1.68510800 -0.17376600

H 2.67616600 -1.61313600 -0.46042900

H 2.01419600 -1.18230000 1.13646200

G^0 = -308.867712 hartrees

DME *gtg* (DMSO – SMD, B3LYP-GD3BJ/6-311++G**)

0 1

C -2.35819500 0.76561200 0.20309000

H -1.84524400 1.18320500 1.07708100

H -2.27783800 1.47887600 -0.62778900

H -3.41187000 0.62580800 0.44999300

O -1.84424600 -0.50887400 -0.16775400

C -0.48420300 -0.47255900 -0.59248100

H -0.31577000 0.39900600 -1.23707300

H -0.31947400 -1.37421400 -1.18753800

C 0.48420300 -0.47255900 0.59248000

H 0.31947400 -1.37421600 1.18753700

H 0.31577000 0.39900400 1.23707400

O 1.84424600 -0.50887500 0.16775400

C 2.35819400 0.76561200 -0.20308900

H 2.27783600 1.47887700 0.62778900

H 3.41187000 0.62580900 -0.44999100

H 1.84524500 1.18320400 -1.07708200

G^0 = -308.866192 hartrees

DME *gtg'* (DMSO – SMD, B3LYP-GD3BJ/6-311++G**)

0 1

C -2.62174600 -0.36898800 0.08263100

H -2.32151800 -1.35649800 -0.28556300

H -2.72350200 -0.41466700 1.17494900

H -3.58892200 -0.11373000 -0.35346200

O -1.70994700 0.65405200 -0.30215900

C -0.43111200 0.54744500 0.31666800

H -0.54132000 0.35343100 1.39249600

H 0.04171900 1.52327200 0.19129800

C 0.43110900 -0.54744500 -0.31665300

H 0.54131200 -0.35343600 -1.39248200

H -0.04171800 -1.52327400 -0.19127800

O 1.70994700 -0.65404300 0.30217000

C 2.62174900 0.36898100 -0.08265200

H 2.32151500 1.35650700 0.28549700

H 3.58892000 0.11374300 0.35346400

H 2.72351800 0.41461400 -1.17497000

G^0 = -308.865720 hartrees

DME *ggg* (DMSO – SMD, B3LYP-GD3BJ/6-311++G**)

0 1

C 2.26538900 -0.46425800 -0.38440900

H 1.78311700 -0.71228500 -1.33684800

H 2.83306300 0.46823200 -0.50772300

H 2.95650300 -1.26787800 -0.12467300

O 1.33065800 -0.35975200 0.68023800

C 0.48169600 0.77796900 0.59125000

H 1.08299000 1.69768400 0.52322100

H -0.06578100 0.81000400 1.53500200

C -0.48169600 0.77796800 -0.59125200

H 0.06578100 0.81000000 -1.53500400

H -1.08299000 1.69768200 -0.52322500

O -1.33065800 -0.35975300 -0.68023700

C -2.26538900 -0.46425700 0.38441000

H -2.83306400 0.46823300 0.50772100

H -2.95650300 -1.26787800 0.12467600

H -1.78311700 -0.71228100 1.33685000

G^0 = -308.866301 hartrees

DME *ggg'* (DMSO – SMD, B3LYP-GD3BJ/6-311++G**)

0 1

C -1.93734100 -0.79630400 0.39676900

H -1.23101000 -1.18618600 1.13864400

H -2.64622000 -0.12784500 0.90380700

H -2.48617800 -1.63444900 -0.03557300

O -1.28357500 -0.13013300 -0.67461800

C -0.63921900 1.08174300 -0.28958600

H -1.34331800 1.72279000 0.26312200

H -0.37744000 1.58585300 -1.22313000

C 0.61633000 0.91667100 0.55503700

H 0.40455900 0.37428200 1.48516000

H 0.95519200 1.92078000 0.82869500

O 1.70058600 0.30013400 -0.13385700

C 1.67869900 -1.12313400 -0.09828300

H 0.82434500 -1.53239500 -0.64573600

H 2.60277600 -1.46663000 -0.56717100

H 1.65039600 -1.49007000 0.93635400

G^0 = -308.866141 hartrees

3*O* tt_nt (DMSO – SMD, B3LYP-GD3BJ/6-311++G**)

0 1

C 4.72467900 -0.37993700 0.00011100

H 4.77385500 -1.01800600 0.89261700

H 4.77388300 -1.01842500 -0.89209400

H 5.57855800 0.29918100 -0.00003600

O 3.54901000 0.41587100 -0.00009200

C 2.36887400 -0.37261100 0.00006300

H 2.33815100 -1.01761400 0.88941000

H 2.33807900 -1.01785900 -0.88910500

C 1.18065800 0.56919600 -0.00002200

H 1.21025400 1.21329600 -0.88945400

H 1.21024700 1.21345100 0.88929700

O 0.00000000 -0.22022100 0.00004100

C -1.18065800 0.56919600 0.00003400

H -1.21021200 1.21342200 -0.88930800

H -1.21028900 1.21332500 0.88944400

C -2.36887400 -0.37261100 -0.00006600

H -2.33811700 -1.01782600 0.88912700

H -2.33811300 -1.01764800 -0.88938800

O -3.54901000 0.41587100 0.00000900

C -4.72467900 -0.37993700 -0.00009100

H -4.77387800 -1.01831400 0.89219400

H -4.77386000 -1.01811700 -0.89251700

H -5.57855800 0.29918100 -0.00002500

G^0 = -462.704239 hartrees

3*O* *tg*$_n$*t* (DMSO – SMD, B3LYP-GD3BJ/6-311++G**)

0 1

C -3.91860400 -1.17605400 0.22797800

H -4.74196900 -0.63744100 -0.26157100

H -3.62025700 -2.01648000 -0.41368800

H -4.26930900 -1.56668200 1.18457100

O -2.82383500 -0.31539000 0.49562500

C -2.29781800 0.26211600 -0.69170300

H -3.06841300 0.87185500 -1.18602000

H -1.98948600 -0.52566100 -1.39383500

C -1.12106000 1.14635400 -0.36458000

H -1.38674700 1.83276900 0.45227300

H -0.88179500 1.75343600 -1.24987800

O 0.00000000 0.35420700 0.00000000

C 1.12106000 1.14635400 0.36458000

H 0.88179500 1.75343600 1.24987800

H 1.38674700 1.83276900 -0.45227300

C 2.29781800 0.26211600 0.69170300

H 1.98948600 -0.52566100 1.39383500

H 3.06841300 0.87185600 1.18602000

O 2.82383500 -0.31539000 -0.49562500

C 3.91860400 -1.17605400 -0.22797800

H 3.62025700 -2.01648000 0.41368800

H 4.74196900 -0.63744100 0.26157000

H 4.26930900 -1.56668300 -1.18457100

G^0 = -462.705642 hartrees

4O tt$_n$t (DMSO – SMD, B3LYP-GD3BJ/6-311++G**)

0 1
C -6.50998500 0.27959900 0.00002400
H -6.57808500 0.91591300 0.89252600
H -6.57806600 0.91637700 -0.89214900
H -7.34332300 -0.42457300 -0.00016900
O -5.31106000 -0.48079100 -0.00015900
C -4.15528400 0.34301900 0.00005000
H -4.14411900 0.98863400 0.88940400
H -4.14398500 0.98889900 -0.88911200
C -2.93894100 -0.56217600 0.00000000
H -2.94863100 -1.20678100 -0.88946900
H -2.94867200 -1.20693800 0.88935500
O -1.78289000 0.26314700 0.00009800
C -0.57877800 -0.48961800 0.00010100
H -0.52890000 -1.13250600 -0.88924200
H -0.52883600 -1.13240100 0.88951800
C 0.57877800 0.48961800 0.00000200
H 0.52887400 1.13253700 0.88932300
H 0.52886200 1.13237100 -0.88943700
O 1.78289000 -0.26314700 0.00006200
C 2.93894200 0.56217600 -0.00003300
H 2.94867700 1.20692700 0.88932900
H 2.94862600 1.20679200 -0.88949400
C 4.15528400 -0.34301900 0.00000000
H 4.14407700 -0.98870200 0.88930500
H 4.14402700 -0.98883100 -0.88921100
O 5.31106000 0.48079100 -0.00009200
C 6.50998500 -0.27959900 -0.00007000
H 6.57805100 -0.91620900 -0.89236400
H 6.57810000 -0.91608100 0.89231100
H 7.34332300 0.42457300 -0.00014400
G^0 = -616.540445 hartrees

4*O tg$_n$t* (DMSO – SMD, B3LYP-GD3BJ/6-311++G**)

0 1
C -5.04417900 -1.41497900 0.81994300
H -5.77976700 -1.64687000 0.03706200
H -4.47276100 -2.32621000 1.04385200
H -5.57256600 -1.09713000 1.72019900
O -4.19032100 -0.35211000 0.42977600
C -3.46224700 -0.66097500 -0.75109500
H -4.15695000 -0.82938600 -1.58714300
H -2.87781200 -1.58111000 -0.60811100
C -2.54324100 0.47884800 -1.11109000
H -3.10240900 1.42564200 -1.10272600
H -2.16983000 0.31782500 -2.13291100
O -1.45613100 0.53902700 -0.19909200
C -0.57766300 1.61801800 -0.48441700
H -1.11246900 2.57447200 -0.39128100
H -0.20072100 1.54195600 -1.51451000
C 0.57766300 1.61801800 0.48441700
H 0.20072100 1.54195700 1.51451000
H 1.11246900 2.57447200 0.39128000
O 1.45613100 0.53902700 0.19909200
C 2.54324100 0.47884800 1.11109000
H 2.16983000 0.31782600 2.13291100
H 3.10240900 1.42564200 1.10272600
C 3.46224700 -0.66097500 0.75109500
H 4.15695000 -0.82938500 1.58714300
H 2.87781300 -1.58111000 0.60811100
O 4.19032100 -0.35211000 -0.42977600
C 5.04417800 -1.41497900 -0.81994300
H 4.47276000 -2.32621000 -1.04385200
H 5.77976700 -1.64687100 -0.03706200
H 5.57256500 -1.09713000 -1.72019900
G^0 = -616.542593 hartrees

5*O tt$_n$t* (DMSO – SMD, B3LYP-GD3BJ/6-311++G**)

0 1
C 8.27295900 -0.41690200 0.00013600
H 8.32232200 -1.05501700 0.89259100
H 8.32234000 -1.05528700 -0.89212500
H 9.12659000 0.26252400 0.00004200
O 7.09698400 0.37852400 0.00000400
C 5.91713900 -0.41038300 0.00011000
H 5.88658900 -1.05541400 0.88944300
H 5.88657400 -1.05563300 -0.88906300
C 4.72864100 0.53105300 0.00000200
H 4.75778800 1.17507900 -0.88946000
H 4.75777200 1.17526500 0.88933000
O 3.54815000 -0.25892600 0.00007400
C 2.36734300 0.52987800 0.00001000
H 2.33679400 1.17386100 -0.88941100
H 2.33675400 1.17394700 0.88936700
C 1.18072700 -0.41390600 0.00003000
H 1.21096200 -1.05783100 0.88947500
H 1.21097400 -1.05788700 -0.88937400
O 0.00000000 0.37529400 -0.00000300
C -1.18072700 -0.41390600 0.00000200
H -1.21097800 -1.05783800 0.88944100
H -1.21095800 -1.05787900 -0.88940800
C -2.36734300 0.52987800 -0.00003300
H -2.33678500 1.17392300 0.88934300
H -2.33676300 1.17388500 -0.88943500
O -3.54815000 -0.25892600 -0.00003000
C -4.72864100 0.53105300 -0.00005600
H -4.75776400 1.17516500 -0.88945700
H -4.75779600 1.17517900 0.88933400
C -5.91713900 -0.41038300 -0.00006900
H -5.88658400 -1.05553200 0.88917800
H -5.88658000 -1.05551500 -0.88932800
O -7.09698400 0.37852400 -0.00006500
C -8.27295900 -0.41690200 -0.00012100
H -8.32231400 -1.05511500 -0.89250700
H -8.32234900 -1.05519000 0.89220900
H -9.12659000 0.26252400 -0.00010900
G^0 = -770.375986 hartrees

5*O* *tg*$_n$*t* (DMSO – SMD, B3LYP-GD3BJ/6-311++G**)

0 1
C 6.48611600 0.78725000 1.22297900
H 7.07611400 1.62340700 0.82242600
H 5.95375600 1.13287700 2.11968200
H 7.16212700 -0.02260900 1.50173500
O 5.58706600 0.27621800 0.25274600
C 4.66442700 1.26283400 -0.18931900
H 5.20369200 2.09480600 -0.66559800
H 4.10407600 1.66957000 0.66463900
C 3.70882400 0.67065600 -1.19404700
H 4.27071200 0.13085300 -1.96997400
H 3.16265900 1.49032200 -1.68329300
O 2.79663600 -0.20387300 -0.54589700
C 1.90277200 -0.81998800 -1.46175500
H 2.46385500 -1.43649400 -2.17917100
H 1.35293900 -0.05695700 -2.03126100
C 0.93059700 -1.70612000 -0.72450400
H 1.47661100 -2.37386600 -0.04261200
H 0.40307500 -2.33250600 -1.45861800
O -0.00003700 -0.91470900 -0.00038900
C -0.93052400 -1.70650700 0.72349100
H -0.40285800 -2.33319000 1.45725000
H -1.47658600 -2.37397800 0.04137100
C -1.90264900 -0.82078600 1.46129700
H -2.46359300 -1.43768000 2.17849000
H -1.35278100 -0.05800100 2.03110000
O -2.79667700 -0.20426600 0.54587300
C -3.70904200 0.66962600 1.19463300
H -3.16302700 1.48892700 1.68465900
H -4.27099300 0.12910900 1.97001800
C -4.66453900 1.26260400 0.19028000
H -4.10408800 1.67018800 -0.66320800
H -5.20397500 2.09408000 0.66723200
O -5.58699900 0.27630300 -0.25286200
C -6.48599200 0.78819300 -1.22270300
H -7.07614000 1.62387800 -0.82138900
H -5.95356000 1.13478000 -2.11899200
H -7.16186700 -0.02146900 -1.50235200
G^0 = -770.378443 hartrees

6*O* tt_nt (DMSO – SMD, B3LYP-GD3BJ/6-311++G**)

0 1
C -10.05149300 0.34076300 -0.00010600
H -10.10914500 0.97809400 0.89239700
H -10.10907500 0.97849500 -0.89232700
H -10.89610400 -0.34977000 -0.00029500
O -8.86536300 -0.43940400 -0.00023300
C -7.69581900 0.36470200 -0.00001600
H -7.67367600 1.00995100 0.88936600
H -7.67352600 1.01021600 -0.88920300
C -6.49533900 -0.56146400 -0.00005700
H -6.51603500 -1.20570500 -0.88955400
H -6.51611800 -1.20589700 0.88929800
O -5.32539200 0.24404500 0.00008300
C -4.13418400 -0.52890500 0.00007900
H -4.09492100 -1.17237600 -0.88933600
H -4.09492200 -1.17239200 0.88948200
C -2.96076300 0.43126500 0.00008800
H -2.99981000 1.07469000 0.88951400
H -2.99977700 1.07466700 -0.88935500
O -1.76935200 -0.34171400 0.00011900
C -0.59969200 0.46377100 0.00012400
H -0.57826600 1.10797100 0.88957900
H -0.57827200 1.10799300 -0.88931400
C 0.59969200 -0.46377100 0.00010600
H 0.57827000 -1.10799900 0.88954000
H 0.57826800 -1.10796300 -0.88935400
O 1.76935200 0.34171400 0.00012100
C 2.96076300 -0.43126500 0.00006800
H 2.99976700 -1.07465700 -0.88938200
H 2.99981900 -1.07470000 0.88948600
C 4.13418400 0.52890500 0.00005900
H 4.09491900 1.17239500 0.88946000
H 4.09492400 1.17237300 -0.88935800
O 5.32539200 -0.24404500 0.00007200
C 6.49533900 0.56146400 -0.00011000
H 6.51604100 1.20565100 -0.88964500
H 6.51611200 1.20595100 0.88920700
C 7.69581900 -0.36470200 -0.00000300
H 7.67366200 -1.00990600 0.88941200
H 7.67354000 -1.01026000 -0.88915800
O 8.86536300 0.43940400 -0.00024200
C 10.05149300 -0.34076300 -0.00014200
H 10.10905900 -0.97848600 -0.89237100
H 10.10916100 -0.97810400 0.89235300
H 10.89610400 0.34977000 -0.00033900
G^0 = -924.212081 hartrees

6*O* *tg*$_n$*t* (DMSO – SMD, B3LYP-GD3BJ/6-311++G**)

0 1
C 7.98421600 1.03488400 0.40257800
H 8.60204100 1.35495100 -0.44810600
H 7.52002000 1.92364900 0.85154700
H 8.62390500 0.55512900 1.14504600
O 7.00775200 0.08635500 0.00541700
C 6.12482800 0.61203200 -0.97651500
H 6.68894700 0.88704800 -1.87990400
H 5.63174200 1.51886900 -0.59808000
C 5.08830700 -0.41668900 -1.35183600
H 5.57903400 -1.37254300 -1.58553400
H 4.56870600 -0.07473600 -2.25881400
O 4.15836200 -0.58538700 -0.29183200
C 3.17738100 -1.56795400 -0.59125700
H 3.65773700 -2.54455800 -0.74952500
H 2.64056000 -1.30345900 -1.51364900
C 2.20094400 -1.69353500 0.55089600
H 2.74741000 -1.81337700 1.49743700
H 1.59505100 -2.59805700 0.39499700
O 1.36213900 -0.54888100 0.60654800
C 0.44262400 -0.61036900 1.68725600
H 0.98583500 -0.64864800 2.64273400
H -0.17351800 -1.51806800 1.61377500
C -0.44262400 0.61033300 1.68726800
H -0.98583600 0.64859100 2.64274700
H 0.17351800 1.51803300 1.61380800
O -1.36213700 0.54886700 0.60655800
C -2.20094300 1.69352200 0.55092900
H -1.59505000 2.59804700 0.39504900
H -2.74740900 1.81334500 1.49747300
C -3.17738000 1.56796400 -0.59122600
H -2.64055900 1.30348600 -1.51362400
H -3.65773500 2.54457100 -0.74947500
O -4.15836200 0.58539200 -0.29182000
C -5.08830600 0.41671400 -1.35182900
H -5.57903100 1.37257200 -1.58551000
H -4.56870500 0.07477600 -2.25881100
C -6.12482800 -0.61201200 -0.97652600
H -6.68894600 -0.88701200 -1.87992100
H -5.63174500 -1.51885600 -0.59810700
O -7.00775300 -0.08635100 0.00541300
C -7.98422000 -1.03488500 0.40255500
H -7.52002700 -1.92365900 0.85150900
H -8.60204400 -1.35493500 -0.44813600
H -8.62390900 -0.55514200 1.14503100
G^0 = -924.214902 hartrees

7*O* tt_nt (DMSO – SMD, B3LYP-GD3BJ/6-311++G**)

0 1
C 11.82038600 -0.43122100 0.00013600
H 11.87014200 -1.06906900 0.89274500
H 11.87013700 -1.06973300 -0.89199900
H 12.67345300 0.24883500 -0.00012100
O 10.64402800 0.36359900 -0.00015600
C 9.46447600 -0.42573800 0.00013500
H 9.43416400 -1.07052600 0.88961300
H 9.43407900 -1.07105700 -0.88895600
C 8.27590900 0.51565900 -0.00009300
H 8.30488400 1.15946500 -0.88967400
H 8.30490200 1.15992200 0.88915700
O 7.09570600 -0.27473900 0.00012200
C 5.91460400 0.51358800 -0.00001600
H 5.88371700 1.15738900 -0.88951700
H 5.88367200 1.15763500 0.88930600
C 4.72867600 -0.43110100 0.00008700
H 4.75922100 -1.07485300 0.88960300
H 4.75921100 -1.07504000 -0.88929300
O 3.54761400 0.35761900 0.00001000
C 2.36722500 -0.43203000 0.00009100
H 2.33708500 -1.07580000 0.88960600
H 2.33709000 -1.07599100 -0.88928600
C 1.18062600 0.51181000 -0.00001400
H 1.21083900 1.15577400 0.88935300
H 1.21084300 1.15557900 -0.88952200
O 0.00000000 -0.27754100 0.00006900
C -1.18062600 0.51181000 -0.00001800
H -1.21082500 1.15560100 -0.88951000
H -1.21085700 1.15575200 0.88936500
C -2.36722500 -0.43203000 0.00004400
H -2.33706000 -1.07586800 0.88950900
H -2.33711500 -1.07592200 -0.88938200
O -3.54761400 0.35761900 0.00005600
C -4.72867600 -0.43110100 -0.00007200
H -4.75921700 -1.07480000 -0.88962700
H -4.75921600 -1.07509400 0.88927000
C -5.91460400 0.51358800 0.00008400
H -5.88372100 1.15733500 0.88962400
H -5.88366900 1.15768900 -0.88919900
O -7.09570600 -0.27473900 -0.00010700
C -8.27590900 0.51565900 0.00004800
H -8.30487900 1.15988800 -0.88922700
H -8.30490600 1.15949800 0.88960500
C -9.46447600 -0.42573800 -0.00017600
H -9.43411100 -1.07059300 -0.88960400
H -9.43413200 -1.07099000 0.88896500
O -10.64402800 0.36359900 -0.00001200
C -11.82038600 -0.43122100 -0.00021400
H -12.67345300 0.24883500 -0.00004300
H -11.87013900 -1.06917600 -0.89274700
H -11.87014100 -1.06962600 0.89199700
G^0 = -1078.047775 hartrees

7*O tg$_n$t* (DMSO – SMD, B3LYP-GD3BJ/6-311++G**)

0 1
C 9.35362500 1.29318400 -0.20746400
H 9.99789400 1.02223800 -1.05552900
H 8.86033800 2.24827300 -0.43436000
H 9.97215100 1.41580200 0.68308500
O 8.40637800 0.27471400 0.06905600
C 7.55421900 0.02559000 -1.04085400
H 8.14843400 -0.31612700 -1.90109600
H 7.03394200 0.94810000 -1.33574700
C 6.54741000 -1.04262900 -0.69643500
H 7.05979300 -1.90895400 -0.25368100
H 6.06062000 -1.37758400 -1.62397100
O 5.57707200 -0.52919800 0.20464700
C 4.63033700 -1.51507000 0.59093000
H 5.13636000 -2.33787000 1.11664400
H 4.13380800 -1.93653500 -0.29497100
C 3.60172200 -0.91501400 1.51591400
H 4.10285100 -0.36643000 2.32644800
H 3.02172200 -1.73142900 1.97044700
O 2.73929000 -0.04881400 0.79287000
C 1.76301100 0.55590600 1.62838500
H 2.25287600 1.17232200 2.39625300
H 1.17016900 -0.21419900 2.14253300
C 0.85488600 1.43956300 0.81103300
H 0.25749600 2.05758100 1.49724300
H 1.45590300 2.11555400 0.18589200
O 0.00000000 0.64661800 0.00000000
C -0.85488600 1.43956300 -0.81103400
H -0.25749600 2.05758100 -1.49724400
H -1.45590300 2.11555400 -0.18589300
C -1.76301100 0.55590600 -1.62838500
H -1.17016900 -0.21420000 -2.14253300
H -2.25287700 1.17232100 -2.39625300
O -2.73929000 -0.04881400 -0.79287000
C -3.60172200 -0.91501500 -1.51591300
H -4.10285100 -0.36643100 -2.32644800
H -3.02172200 -1.73143000 -1.97044700
C -4.63033700 -1.51507100 -0.59093000
H -5.13636000 -2.33787100 -1.11664300
H -4.13380800 -1.93653500 0.29497200
O -5.57707200 -0.52919800 -0.20464700
C -6.54741000 -1.04262900 0.69643500
H -6.06062000 -1.37758400 1.62397100
H -7.05979300 -1.90895400 0.25368100
C -7.55421900 0.02559000 1.04085500
H -8.14843400 -0.31612700 1.90109600
H -7.03394100 0.94810100 1.33574600
O -8.40637900 0.27471400 -0.06905600
C -9.35362500 1.29318400 0.20746400
H -9.97215100 1.41580200 -0.68308500
H -9.99789400 1.02223800 1.05552900
H -8.86033800 2.24827300 0.43436000
G^0 = -1078.052652 hartrees
